# Isoforms of Cathepsin B1 in Neurotropic Schistosomula of *Trichobilharzia regenti* Differ in Substrate Preferences and a Highly Expressed Catalytically Inactive Paralog Binds Cystatin

**DOI:** 10.3389/fcimb.2020.00066

**Published:** 2020-02-26

**Authors:** Hana Dvořáková, Roman Leontovyč, Tomáš Macháček, Anthony J. O'Donoghue, Ondřej Šedo, Zbyněk Zdráhal, Charles S. Craik, Conor R. Caffrey, Petr Horák, Libor Mikeš

**Affiliations:** ^1^Department of Parasitology, Faculty of Science, Charles University, Prague, Czechia; ^2^Department of Pharmaceutical Chemistry, School of Pharmacy, University of California, San Francisco, San Francisco, CA, United States; ^3^Central European Institute of Technology, Masaryk University, Brno, Czechia; ^4^Center for Discovery and Innovation in Parasitic Diseases, Department of Pathology, University of California, San Francisco, San Francisco, CA, United States

**Keywords:** peptidase, cathepsin B, processing, substrate specificity, occluding loop, cystatin, helminth, schistosome

## Abstract

Schistosomula (the post-infective stages) of the neurotropic schistosome *Trichobilharzia regenti* possess multiple isoforms of cathepsin B1 peptidase (TrCB1.1-TrCB1.6) with involvement in nutrient digestion. The comparison of substrate preferences of TrCB1.1 and TrCB1.4 showed that TrCB1.4 had a very narrow substrate specificity and after processing it was less effective toward protein substrates when compared to TrCB1.1. Self-processing of both isoforms could be facilitated by sulfated polysaccharides due to a specific binding motif in the pro-sequence. Trans-activation by heterologous enzymes was also successfully employed. Expression profiling revealed a high level of transcription of genes encoding the enzymatically inactive paralogs TrCB1.5 and TrCB1.6. The transcription level of TrCB1.6 was comparable with that of TrCB1.1 and TrCB1.2, the most abundant active isoforms. Recombinant TrCB1.6wt, a wild type paralog with a Cys^29^-to-Gly substitution in the active site that renders the enzyme inactive, was processed by the active TrCB1 forms and by an asparaginyl endopeptidase. Although TrCB1.6wt lacked hydrolytic activity, endopeptidase, but not dipeptidase, activity could be restored by mutating Gly^29^ to Cys^29^. The lack of exopeptidase activity may be due to other mutations, such as His^110^-to-Asn in the occluding loop and Asp^224^-to-Gly in the main body of the mature TrCB1.6, which do not occur in the active isoforms TrCB1.1 and TrCB1.4 with exopeptidase activity. The catalytically active enzymes and the inactive TrCB1.6 paralog formed complexes with chicken cystatin, thus supporting experimentally the hypothesis that inactive paralogs could potentially regulate the activity of the active forms or protect them from being inhibited by host inhibitors. The effect on cell viability and nitric oxide production by selected immune cells observed for TrCB1.1 was not confirmed for TrCB1.6. We show here that the active isoforms of TrCB1 have different affinities for peptide substrates thereby facilitating diversity in protein-derived nutrition for the parasite. The inactive paralogs are unexpectedly highly expressed and one of them retains the ability to bind cystatins, likely due to specific mutations in the occluding loop and the enzyme body. This suggests a role in sequestration of inhibitors and protection of active cysteine peptidases.

## Introduction

Flukes (Trematoda: Digenea) of the family Schistosomatidae include blood-dwelling flatworms that are pathogenic in birds and mammals, including man. Their infectious juveniles (cercariae) developing in intermediate snail hosts penetrate the skin of the definitive host in the water and enter the blood circulation while transforming to post-infective stages—the schistosomula (Kašný et al., 2016; Řimnáčová et al., 2017). In humans, the invasion of the skin can be accompanied by an allergic reaction called cercarial dermatitis (Horák et al., 2015; Macháček et al., 2018). However, schistosomula of the avian schistosome *Trichobilharzia regenti* use peripheral nerves and the central nervous system (CNS) as a migratory route to the final localization in the nasal mucosa of the duck host (Horák et al., 1998, 1999). The migration through the CNS can be manifested by neuromotor symptoms (leg paralysis and balance disorders) in birds and experimentally infected mice, occasionally leading to death of the host (Horák et al., 1999, 2008).

The migrating schistosomula actively feed on nervous tissue (Lichtenbergová et al., 2011; Leontovyč et al., 2019) and the adults in the nasal cavity ingest blood (Chanová and Horák, 2007). Indeed, both stages require gut-associated peptidases to digest host proteins. In *T. regenti*, isoforms of the cysteine peptidase cathepsin B1 (TrCB1) are expressed in the gut and represent a subset of a larger group of peptidases that are responsible for digestion (Dvořák et al., 2005; Dolečková et al., 2010; Leontovyč et al., 2016, 2019), much like in human *Schistosoma* species (Caffrey et al., 2004) and other helminths (Williamson et al., 2003, 2004; Delcroix et al., 2006; Caffrey et al., 2018). Cathepsin B (IUPAC: EC 3.4.22.1; MEROPS: Clan CA, Family C1) is unique among other papain-like cysteine peptidases due to its ability to act as both an endopeptidase and carboxy-exopeptidase (peptidyl dipeptidase) (Barrett and Kirschke, 1981). The latter activity is enabled due to the presence of an extra structural element termed “occluding loop,” which occupies S′ subsites of the enzyme (Musil et al., 1991; Illy et al., 1997). Cathepsin B is synthesized as an inative precursor and a mature enzyme arises as a consequence of proteolytic removal of the N-terminal pro-peptide that sterically blocks the active site (Mort and Buttle, 1997). Six forms of TrCB1 (TrCB1.1 – TrCB1.6, GenBank: AY648119 – 24) were previously identified (Dvořák et al., 2005). Two of these, namely TrCB1.5 and TrCB1.6, are paralogs possessing a substitution of the catalytic cysteine Cys^29^ for a glycine Gly^29^ (mature TrCB1 numbering). Paralogs with similar single-amino acid mutations in the catalytic site are known for many parasite peptidases (Merckelbach et al., 1994; Caffrey et al., 2000; Holt et al., 2004; He et al., 2005; McCoubrie et al., 2007; Mendoza-Palomares et al., 2008; Pillay et al., 2010; Jedličková et al., 2018). Substitution of the active site cysteine need not always necessarily abolish the enzyme's activity, as shown in cases of Cys-to-Ser nucleophile substitutions (McCoubrie et al., 2007; Pillay et al., 2010), whereas other substitutions are expected to produce loss of peptidase activity, which is the case of TrCB1.5 and 1.6. It has been proposed that inactive paralogs of peptidases might regulate the activity of active forms by competing for substrates or inhibitors (Merckelbach et al., 1994; Holt et al., 2003, 2004; Dvořák et al., 2005). Moreover, they may alter host immune response through the inhibition of host complement pathways by binding the complement components C1q, mannose-binding lectin or properdin (Holt et al., 2004; Bergström et al., 2009; Reynolds et al., 2014). Active cathepsin B1 of *Schistosoma mansoni* (SmCB1) diminished the proinflamatory reaction of activated macrophages (Donnelly et al., 2009), provoked mixed Th1/Th2/Th17 immune response in mice and induced a transient T-helper 17 response in acute schistosome infection (Soloviova et al., 2019), suggesting it has a considerable immunogenic capacity. As for *T*. *regenti*, an inflammation-promoting activity was noticed in case of active TrCB1.1 which triggered the production of nitric oxide and proinflammatory cytokines interleukin-6 and tumor necrosis factor α by astrocytes and/or microglia (Macháček et al., 2016), i.e., the cells activated in mice infected experimentally by *T. regenti* (Lichtenbergová et al., 2011). However, no specific immunomodulatory mechanisms were tested so far with inactive peptidase paralogs of helminths.

Although the amino acid sequence identity among the six forms of TrCB1 is high (98 – 99% between the active forms TrCB1.1–TrCB1.4) (Dvořák et al., 2005), it seems that even a small change (a single amino acid substitution) in the sequences, especially in the S2 subsite, is likely to alter substrate specificity. Thus, the presence of multiple forms of a particular enzyme may indicate an adaptation of the parasite to physiological changes in the course of migration through the nervous tissue and final settlement in the nasal mucosa, enabling digestion of various host components.

In this study, we performed an in-depth comparison of substrate preferences of the two most divergent active homologs, TrCB1.1 and TrCB1.4, using positional scanning synthetic combinatorial peptide library and mass spectrometry. We also evaluated the ratios of relative expression levels of all six TrCB1 homologs using transcript-specific Illumina reads and stage-specific transcriptomes of *T*. *regenti* cercariae and schistosomula.We attempted to identify the possible biological roles of the cathepsin B paralogs employing heterologously expressed wild type TrCB1.6wt and its site-directed mutant TrCB1.6G/C (retrograde Gly^29^-to-Cys^29^ mutation). Various methods of enzymatic processing (pro-peptide sequence removal) were applied to all four zymogens included in this study. Finally, we assessed the effects of TrCB1.6wt paralog on cell viability and nitric oxide production using murine astrocytes, microglia and macrophages.

## Results

### Expression of TrCB1 Forms

All recombinant pro-TrCB1 forms produced in *Pichia pastoris* X-33 were hyperglycosylated by the yeast. Each pro-enzyme migrated as a fuzzy band around 50 kDa on SDS-PAGE. After enzymatic deglycosylation and subsequent purification, they occurred as prominent ≈36 kDa bands (Supplementary Figure 1), which corresponds with their theoretical molecular weight (MW) (Table 1). N-terminal amino acid sequences NEMQF of pro-TrCB1.6wt and pro-TrCB1.6G/C, and ENEIQ of pro-TrCB1.1 and pro-TrCB1.4 corresponded to the beginning of the enzymes' predicted pro-sequences. In addition, a few minor bands of lower MW appeared in fractions of deglycosylated purified pro-TrCB1.6G/C, pro-TrCB1.1, and pro-TrCB1.4 (Supplementary Figure 1).

**Table 1:** Theoretical molecular weight and number of potential N-glycosylation sites of pro-enzymes/mature TrCB1 forms.

	**Pro-enzyme (kDa)**	**Mature enzyme (kDa)**	**N-gly sites (pro-sequence/mature enzyme)**
TrCB1.1	36.4	28.5	1/1
TrCB1.4	36.5	28.6	1/1
TrCB1.6wt	36.0	28.2	1/2
TrCB1.6G/C	36.1	28.3	1/2

### Processing of Recombinant *Pichia*-Derived Pro-enzymes

**Figure 1:**
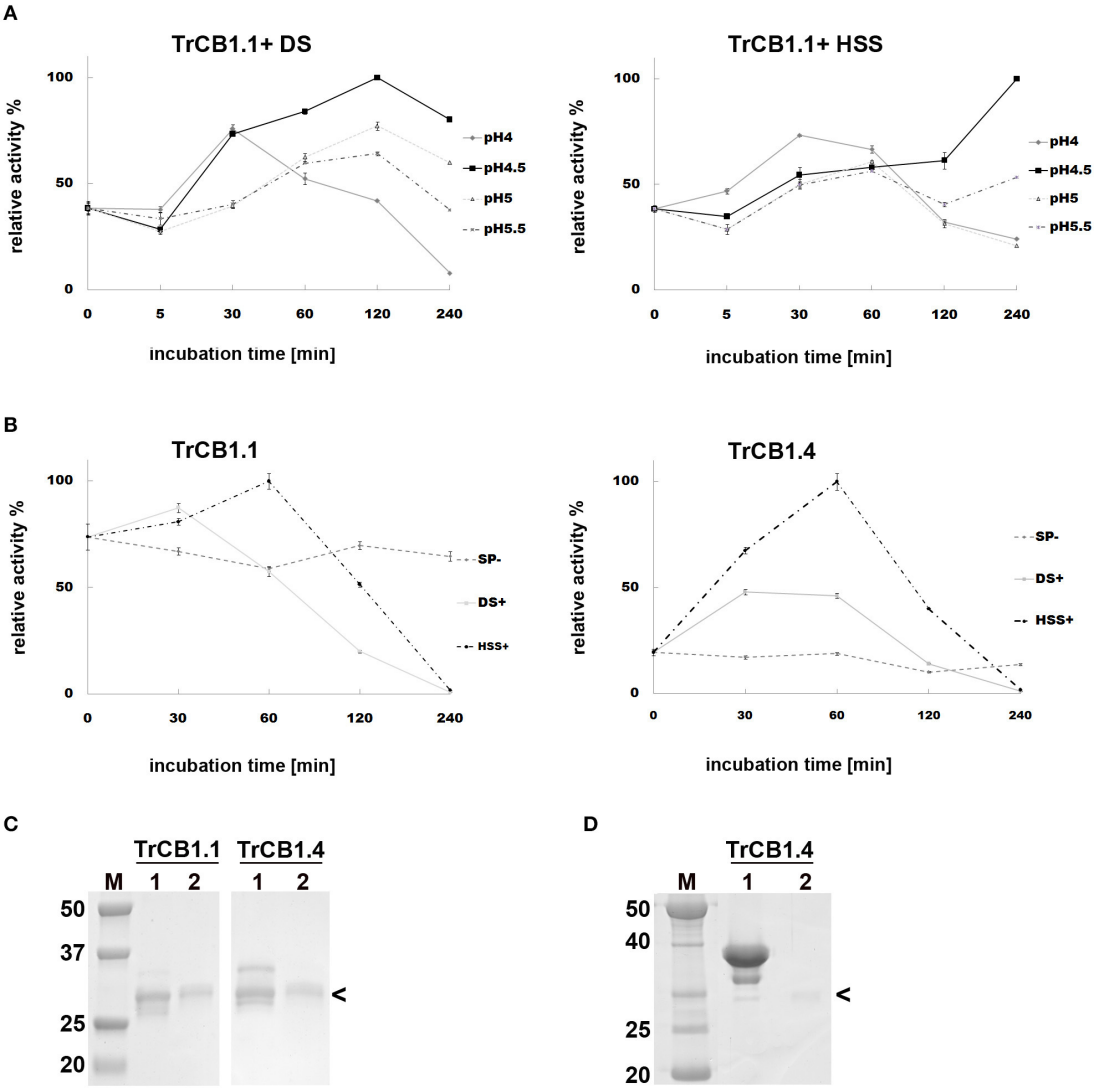
Effect of sulfated polysaccharides on the autocatalytic activation of pro-TrCB1.1 and pro-TrCB1.4. Peptidolytic activities of TrCB1 forms generated after activation were determined in a kinetic assay with Z-Phe-Arg-AMC. The mean values ± standard deviation (SD) of two triplicate assays are expressed as percentage of relative activity in the sample at specified time points. **(A)** pro-TrCB1.1 was incubated in the presence of 10 μg/ml dextran sulfate (DS) or heparin sodium salt (HSS) at various pH values. **(B)** pro-TrCB1.1 and pro-TrCB1.4 were incubated at pH 4.5 in the presence or absence of 20 μg/ml sulfated polysaccharides (SP). **(C)** SDS-PAGE of (pro-)TrCB1.1 and (pro-)TrCB1.4 incubated in the presence of 10 μg/ml DS at pH 4.5 for 30 min (lanes 2). Lanes 1 contain the pro-enzyme without DS. **(D)** SDS-PAGE of (pro-)TrCB1.4 incubated in the presence of 10 μg/ml HSS at pH 4.5 for 30 min (lane 2). Lane 1 contains the pro-enzyme without HSS. M, markers of molecular size (kDa). Arrowheads point to processed enzymes, which were rapidly autodegraded under the experimental conditions. A gel for TrCB1.1 with HSS is not available.

**Figure 2:**
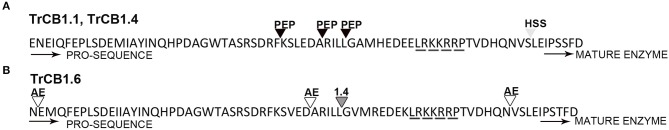
Cleavage sites in the processed pro-sequences of TrCB1 forms. **(A)** cleavage sites determined by Edman degradation in pepsin-processed pepTrCB1.1 and pepTrCB1.4 (black triangles); cleavage site in TrCB1.4 auto-processing product resulted after 1-h incubation in the presence of heparin sodium salt (light gray triangle). **(B)** cleavage sites in rIrAE-activated TrCB1.6wt resulted after 1-h incubation at pH 5 (empty triangles); cleavage site in TrCB1.6wt after processing with pepTrCB1.4 (dark gray triangle). “Heparin-binding” (Horn et al., 2011) motif responsible for glycosaminoglycan binding to the pro-sequences is underlined by a dashed line.

**(A)** Incubation of pro-TrCB1.1 and pro-TrCB1.4 at pH 4 or pH 5.5 for up to 16 h resulted in a decrease in abundance of the pro-protein but no detectable appearance of the lower MW mature enzymes (an example is presented in Supplementary Figure 2). However, addition of 20 μg/ml sulfated polysaccharides (SP)—dextran sulfate (DS) in the case of TrCB1.1 and TrCB1.4 or heparin sodium salt (HSS) with TrCB1.4—resulted in reliable production of ≈30 kDa processed enzymes at pH 4.5 (Figures 1C,D). N-terminal sequencing revealed a L^88^EIPS sequence (pro-TrCB1 numbering) in TrCB1.4 that was processed in the presence of HSS (Figure 2A). The detected cleavage site was two amino acid residues upstream from the predicted beginning of the mature enzyme (Dvořák et al., 2005). Further efforts to determine N-terminal residues in ≈30 kDa SP-treated TrCB1.1 and DS-treated dsTrCB1.4 failed. Incubation of pro-enzymes with both SPs resulted in higher activity against the fluorogenic substrate Z-Phe-Arg-AMC, especially at pH 4.5 (Figure 1A). However, a rapid decline of the activity due to enzyme autodegradation could be observed between ~1 and 4 h (Figure 1B), even when stored on ice.

**Figure 3:**
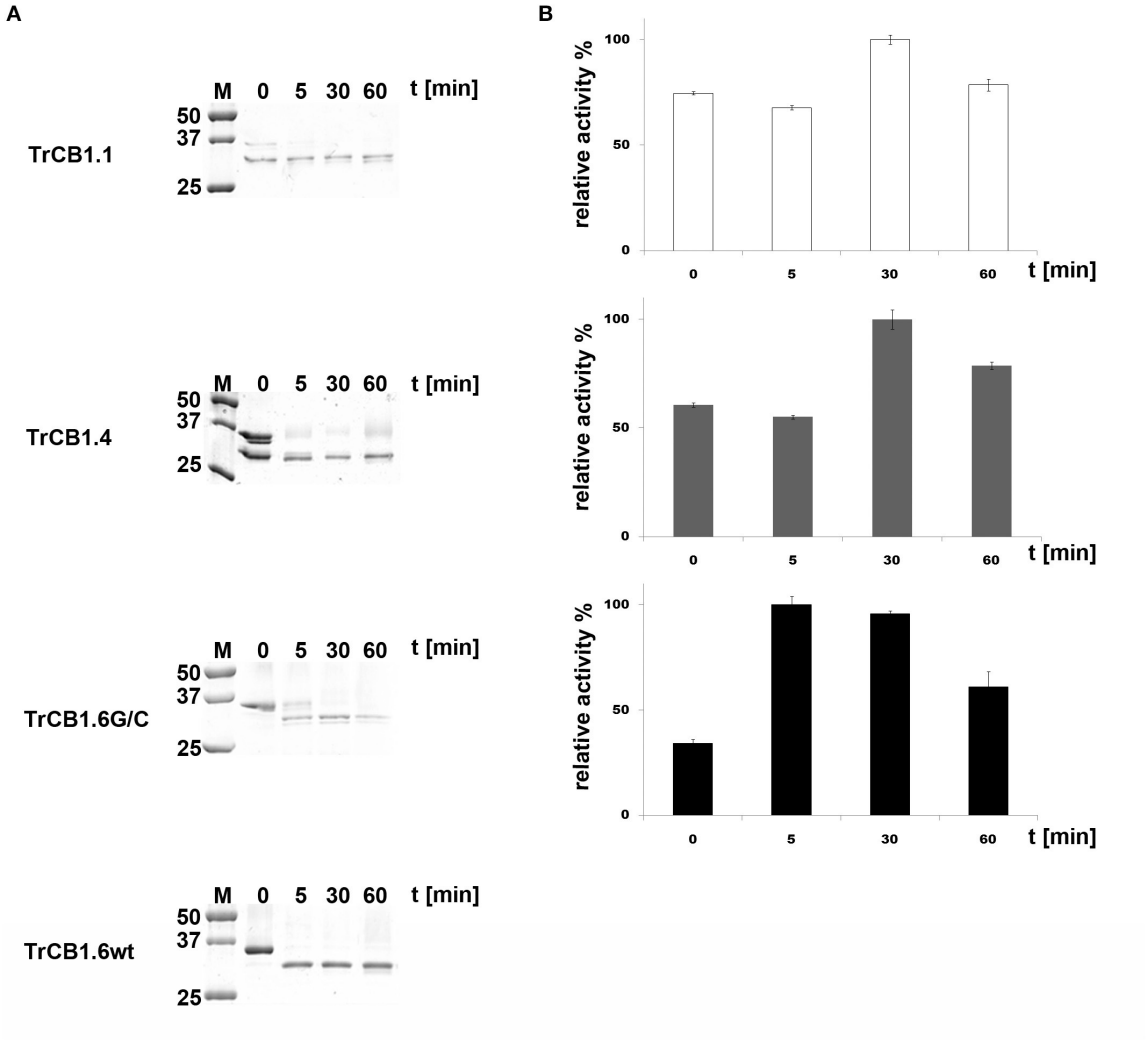
Trans-processing of the TrCB1 zymogens by pepsin. TrCB1 forms were treated with pepsin immobilized on agarose beads at 37°C (TrCB1.6G/C at RT), and the reaction mixtures were analyzed at the indicated time points. **(A)** The processed forms were resolved by SDS-PAGE. M, markers of molecular size. **(B)** Peptidolytic activities of TrCB1.1, TrCB1.4, TrCB1.6G/C generated after processing were measured in a kinetic assay with the substrate Z-Phe-Arg-AMC (25 μM). The results are means ± SD of two triplicate assays and are expressed as percentage of relative activity in the sample.

**Figure 4:**
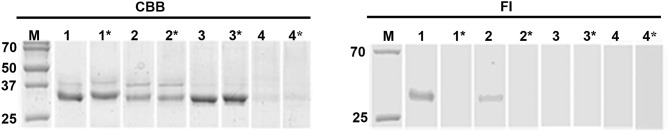
Active site labeling of the pepTrCB1 forms by the cysteine peptidase-specific probe DCG-04. The pepTrCB1 forms were treated with the probe BODIPY green-DCG-04, resolved by SDS-PAGE, and stained with Coomassie Brilliant Blue (CBB) or visualized with a fluorescence imager (Fl). (1) TrCB1.1, (2) TrCB1.4, (3) TrCB1.6wt, (4) TrCB1.6G/C. Samples marked by asterisk were pre-incubated with E-64 inhibitor prior to labeling. M, markers of molecular size (kDa). Note the autodegradation of TrCB1.6G/C (lanes 4 and 4*) during incubation with the probe/inhibitor, resulting in weak protein bands.

**(B)** SDS-PAGE demonstrated that agarose-bound pepsin succesfully processed all pro-enzymes, since new single/double bands of ≈30–32 kDa appeared in the gel (Figure 3A). N-terminal sequencing of pepsin-treated pepTrCB1.1 and pepTrCB1.4 after 1-h incubation revealed that at least 3 products arose due to different cleavage sites within the pro-sequences (Figure 2A), but none of the products started with predicted N-terminal residues of mature enzymes. The pepTrCB1.1, pepTrCB1.4, and pepTrCB1.6G/C had increased activity toward Z-Phe-Arg-AMC compared to corresponding pro-enzymes. Maximum activities were observed within 30 min of incubation in samples of TrCB1.1 and 1.4 and within 5 min in the case of TrCB1.6G/G (Figure 3B). The pepTrCB1.1 and pepTrCB1.4 were stable even after longer (weeks) storage at −25°C. On the other hand, the pepTrCB1.6G/C had very limited stability. The fluorescent active-site probe DCG-04 bound to pepTrCB1.1 and pepTrCB1.4 (Figure 4). This reaction was blocked by the irreversible inhibitor of papain-like cysteine peptidases E-64. No binding of the probe to pepTrCB1.6wt and pepTrCB1.6G/C was observed (Figure 4). Unless otherwise noted, the pepTrCB1 forms (after 30 min incubation with pepsin) were used in subsequent experiments.

**Figure 5:**
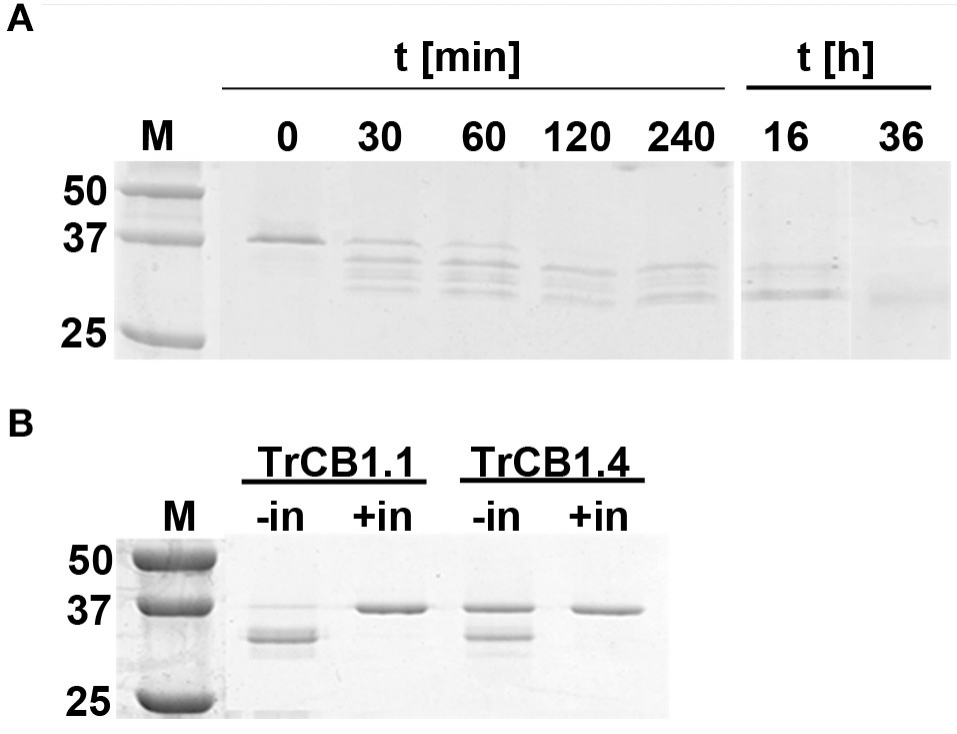
Trans-processing of pro-TrCB1.6wt by IrAE, TrCB1.1, or TrCB1.4. **(A)** The pro-TrCB1.6wt was incubated at pH 5 with purified yeast medium containing activated IrAE for various time periods at 37°C. The TrCB1.6wt processing products were resolved by SDS-PAGE. **(B)** The TrCB1.6wt zymogen was incubated overnight at 37°C with pepTrCB1.1 or pepTrCB1.4 in the presence of dextran sulfate (10 μg/ml). Processing of pro-TrCB1.6wt was visualized by SDS-PAGE, gel stained with Coomassie Brilliant Blue. Processing was inhibited with 10 μM inhibitor of cysteine peptidases E-64. –/+ in denotes absence/presence of the inhibitor. M, markers of molecular size (kDa).

**(C)** Trans-processing of pro-TrCB.6wt by recombinant asparaginyl endopeptidase from the tick *Ixodes ricinus* (IrAE) led to a production of variously processed forms (Figure 5A). All tested pH values gave the same results (Supplementary Figure 3). Three cleavage sites identified by N-terminal sequencing were N^23^/E^24^, D^59^/A^60^, N^85^/V^86^ (pro-TrCB1.6 numbering) (Figure 2B). Prolonged incubation at pH 5.5 (up to 36 h) resulted in the formation of a single ≈30 kDa band, but N-terminal sequencing failed in this case. The intensity of the upper band (TrCB1.6 pro-enzyme) present in the lane at 16 h decreased to undetectable levels compared to time 0 (Figure 5A).

**(D)** Overnight incubation of pro-TrCB1.6wt with pepTrCB1.1 or pepTrCB1.4 in an activation buffer of pH 4.5 supplemented with DS resulted in the conversion of the zymogens to a major band of ≈32 kDa, and a minor band of ca 30 kDa (Figure 5B). Processing was not observed when E-64 (10 μM) was added to the reactions (Figure 5B). The N-terminal sequence G^65^VMRE (pro-TrCB1.6 numbering; Figure 2B) was detected in the more intense ≈32 kDa band after overnight incubation of pro-TrCB1.6wt with pepTrCB1.4, while N-terminal sequencing of the other products was unsuccessful.

### Peptidase Activity and Inhibition Assays of TrCB1.6, TrCB1.1, and TrCB1.4

**Figure 6:**
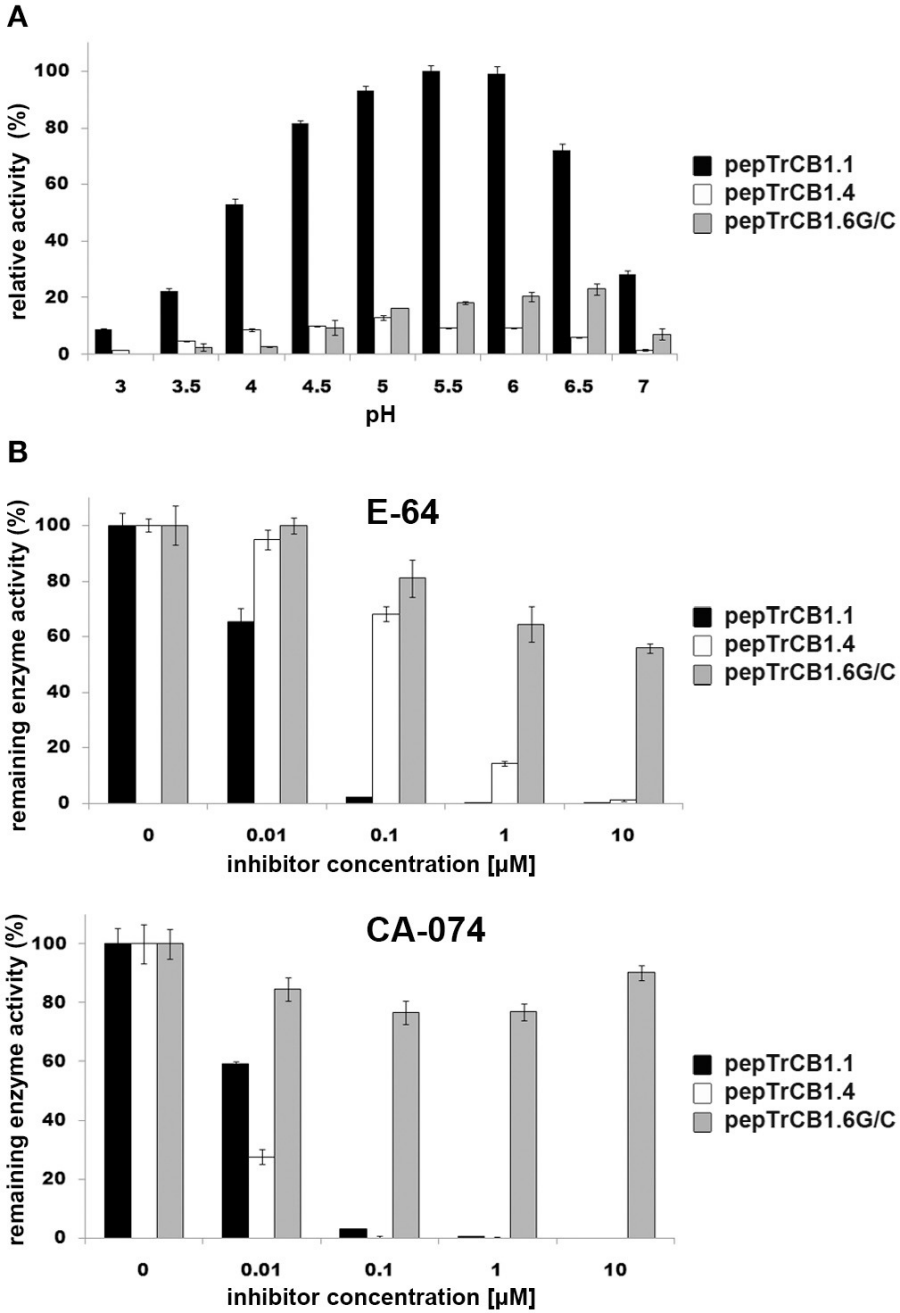
pH optima of the pepTrCB1 forms and effect of inhibitors on enzyme endopeptidase activity. **(A)** The effect of pH on peptidolytic activity of pepTrCB1.1, pepTrCB1.4, pepTrCB1.6G/C toward the fluorogenic peptidyl substrate Z-Phe-Arg-AMC was measured in 50/100 mM CPB (pH range 3–7) with 2 mM DTT. Data are expressed as percentage of relative activity in the sample. **(B)** Effect of cysteine peptidase inhibitors (E-64, CA-074) on the activity of pepTrCB1.1, pepTrCB1.4, pepTrCB1.6G/C toward Z-Phe-Arg-AMC substrate. Inhibition assays were carried out at pH optimum of the particular enzymes. The values are mean ± SD of two triplicate assays.

Assays were performed with two synthetic fluorogenic substrates: Z-Phe-Arg-AMC and Z-Arg-Arg-AMC. The pepTrCB1.6wt had no activity with both substrates (not shown). The activity optimum of pepTrCB1.6G/C with Z-Phe-Arg-AMC occurred at pH 6, i.e., it was shifted more to the neutral value than that of pepTrCB1.1 (pH 5.5) and pepTrCB1.4 (pH 5) (Figure 6A). The pepTrCB1.1 was also active toward Z-Arg-Arg-AMC with a similar optimum at pH 5.5 (not shown). No activity toward this substrate was detected for pepTrCB1.6G/C as well as for pepTrCB1.4.

The activities of pepTrCB1.1 and pepTrCB1.4 were inhibited by E-64 and the cathepsin B-specific irreversible inhibitor CA-074 (Figure 6B). In the case of pepTrCB1.1, <1% of the activity remained after inhibition by both inhibitors at 0.1 μM concentration. Approximately 1% activity of pepTrCB1.4 remained after treatment by 10 μM E-64, while 0.1 μM CA-074 completely suppressed the activity of TrCB1.4. On the other hand, E-64 and CA-074 had little and no effect, respectively, on the activity of pepTrCB1.6G/C (Figure 6B). Even a high concentration (100 μM) of E-64 reduced the activity of pepTrCB1.6G/C only to 34%.

**Figure 7:**
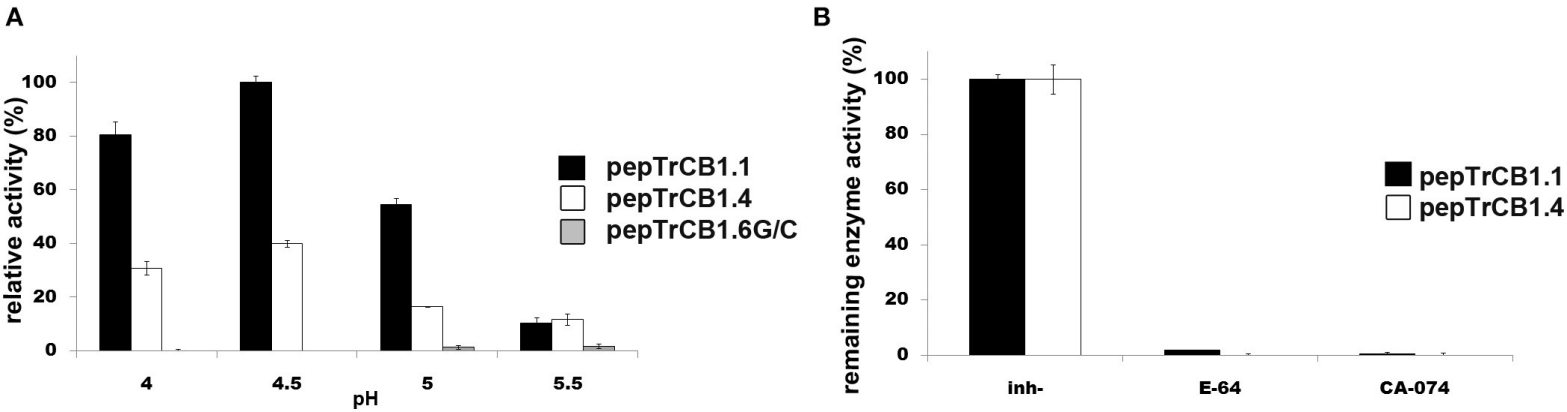
Peptidyl-dipeptidase activity of the pepTrCB1 forms and the effect of inhibitors. **(A)** pepTrCB1 forms were incubated with Bz-Gly-His-Leu substrate in 50/100 mM CPB (pH 4–5.5) containing 2 mM DTT. The appearance of arising free amino groups was monitored by reaction with fluorescamine (0.05 mg/ml). **(B)** The effect of 10μM E-64 and CA-074 on carboxypeptidase activity of pepTrCB1.1 and pepTrCB1.4 assayed at pH optima of the particular enzymes. Values are means of two triplicate assays ± SD.

The peptidyl dipeptidase activity of pepTrCB1 was investigated using Bz-Gly-His-Leu substrate (Figure 7). Both pepTrCB1.1 and pepTrCB1.4 exhibited carboxypeptidase activity against this substrate with an optimum at pH 4.5 (Figure 7A). The activities were efficiently inhibited by 10 μM E-64 and CA-074 (Figure 7B). On the other hand, pepTrCB1.6G/C had very low activity against the exo-substrate at pH 5 and 5.5, and no activity occurred at pH ≤ 5. The pepTrCB1.6wt had no exopeptidase activity at any tested pH (not shown).

Repeated attempts to inhibit endopeptidase activity of activated TrCB1 forms (ds/pepTrCB1.1, ds/pepTrCB1.4, ds/pepTrCB1.6G/C) by chicken egg white cystatin and human cystatins B and C in the presence of Z-Phe-Arg-AMC substrate failed under the conditions used (not shown).

### Formation of Peptidase Complex With Chicken Egg Cystatin

**Figure 8:**
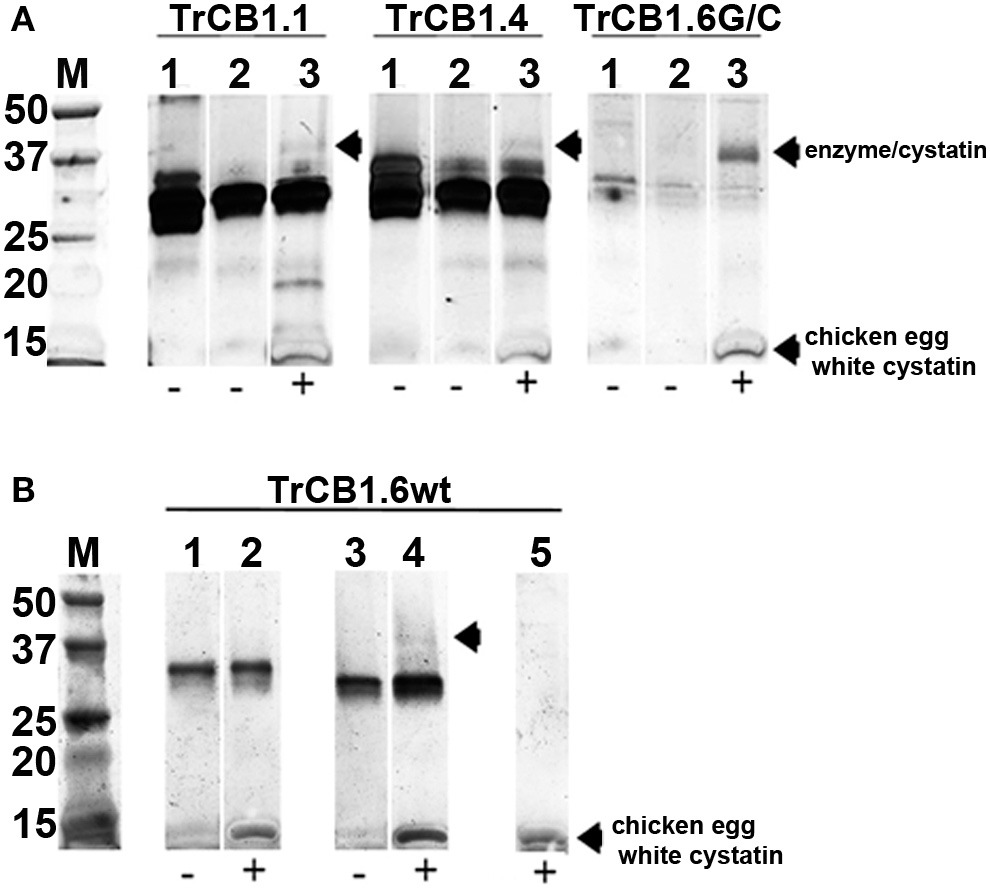
Complex formation between TrCB1 forms and chicken egg white cystatin monitored by SDS-PAGE. **(A)** Auto-activated dsTrCB1.1, dsTrCB1.4, dsTrCB1.6G/C after overnight incubation at 4°C in the presence/absence of chicken egg white cystatin. After incubation, the samples were mixed with non-reducing sample buffer and resolved by SDS-PAGE. Proteins were visualized by silver staining. (1) pro-TrCB1, (2) dsTrCB1 without cystatin, (3) dsTrCB1 with cystatin. Note the autodegradation of TrCB1.6G/C (lanes 1 to 3) happening during incubation, resulting in weak protein bands. **(B)** pepTrCB1.6wt after overnight incubation at 4°C in the presence/absence of cystatin. (1) pro-TrCB1.6wt, (2) pro-TrCB1.6wt with cystatin, (3) pepTrCB1.6wt, (4) pepTrCB1.6wt with cystatin, (5) control lane with cystatin only. Arrows point to peptidase/cystatin complex and/or to cystatin (in section B, lane 5). M, markers of molecular size (kDa).

Overnight incubation at 4°C, but not a 15 min treatment at room temperature (RT) of auto-activated TrCB1 forms (dsTrCB1.1, dsTrCB1.4, dsTrCB1.6G/C) or pepTrCB1.6wt with chicken egg white cystatin resulted in the formation of new weak bands around ≈40 kDa that roughly matched the predicted size of the enzyme/cystatin complex (Figure 8). Only a small percentage of the enzymes bound to cystatin. On the other hand, chicken cystatin seemed to protect TrCB1.6G/C from autodegradation. Binding of human cystatins B and C to the pepsin-processed enzymes was not confirmed within 2 h of incubation at RT (not shown).

### P1–P4 Specificity Profile of TrCB1.1 and TrCB1.4

**Figure 9:**
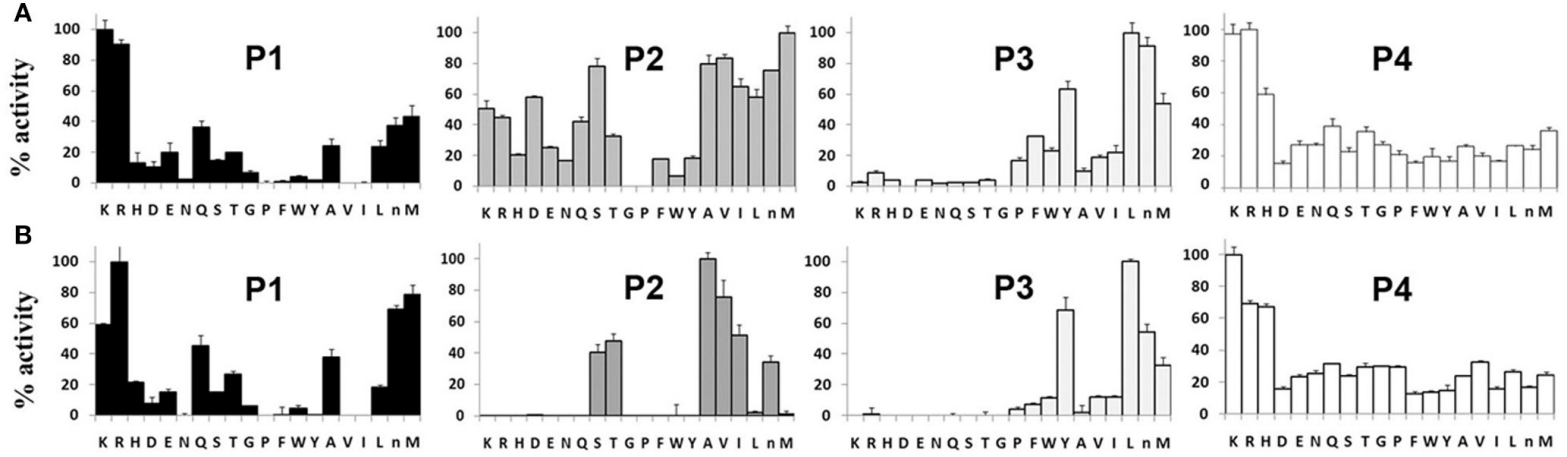
P1–P4 amino acid preferences of pro-TrCB1.1 and pro-TrCB1.4 determined by positional scanning of a synthetic combinatorial library. **(A)** Substrate library of pro-TrCB1.1. **(B)** Substrate library of pro-TrCB1.4. P4-P1 represent the four amino acid side chains recognized by peptidase on the N-terminal side of the cleaved peptide bond. The X-axis indicates the 20 amino acids held constant at each position, designated by the single-letter code (n, norleucine). The Y-axis shows relative preference for the particular amino acid when 100% represents the most preferred amino acid residue.

The preference of TrCB1.1 and TrCB1.4 for amino acid residues at P1 – P4 positions of the substrates was determined using positional scanning synthetic combinatorial library (PS-SCL) (Figure 9). At P1 position, both enzymes preferred small basic amino acids Lys and Arg, but TrCB1.4 showed more distinct inclination to Arg. A stronger preference for Met, the non-natural norleucine (similar unbranched chain structure as Lys), and Lys at this position was also noted for TrCB1.4.

At P2 position, TrCB1.4 exhibited preference for non-polar aliphatic amino acids (Ala, Val, Ile), with overriding preference for Ala. In addition, TrCB1.4 was also able to accommodate polar residues (Ser, Thr and norLeu). TrCB1.1 showed greater promiscuity at P2, with a low preference for large non-polar residues, and could not accept Gly or Pro. However, it was able to hydrolyze substrates with Arg at P2.

Both enzymes displayed similar preferences for residues at P3. The highest preference was recorded for Leu, but the enzymes also well-accepted other amino acid residues (norLeu, Tyr, and Met) at this position. Screening at P4 position revealed a broader substrate specificity, with all amino acid residues accepted by both recombinant enzymes.

### Hydrolysis of Protein Substrates by TrCB1 Forms

**Figure 10:**
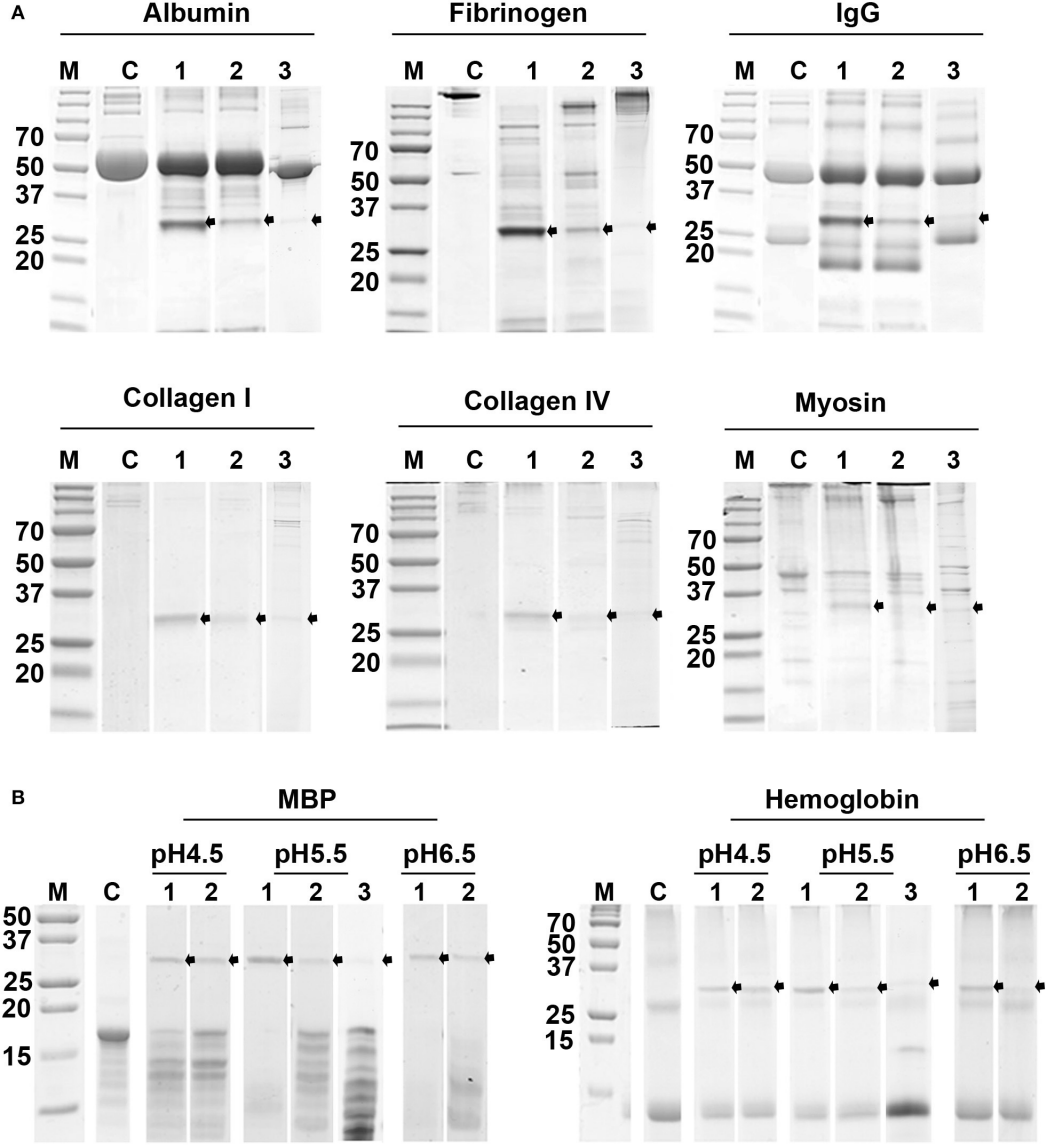
Digestion of selected protein substrates by recombinant TrCB1 forms. The substrates (0.5 mg/ml) were incubated for 6 h with pepTrCB1 forms (1 μg) at 37°C. Aliquots (20 μl) of the resulting hydrolysates were separated by SDS-PAGE and stained with Coomassie Brilliant Blue. **(A)** Incubation of pepTrCB1.1, pepTrCB1.4 and pepTrCB1.6G/C (lanes 1–3, respectively) at pH 5.5 with albumin, fibrinogen, collagen type I, collagen type IV and myosin. **(B)** Incubation of pepTrCB1.1, pepTrCB1.4 and pepTrCB1.6G/C (lanes 1–3, respectively) at pH 4.5–6.5 with myelin basic protein (MBP) and hemoglobin. C, controls (substrates without enzyme). The starting amount of the peptidases was equal in all samples; note the autodegradation of the enzymes under given conditions, resulting in weaker bands especially in TrCB1.6G/C. Arrows indicate the visible peptidase bands in the gels. M, markers of molecular size (kDa).

The pepTrCB1.1 and pepTrCB1.4 were able to degrade all given macromolecular substrates [albumin, IgG, fibrinogen, collagen type I and type IV, myosin, myelin basic protein (MBP) and hemoglobin (Hb)] at pH 5.5 (Figure 10A). While MBP was efficiently hydrolyzed between pH 4.5 and 6.5 with the best result at pH up to 5.5, Hb was poorly degraded only at lower pH by both recombinant enzymes (Figure 10B). The pepTrCB1.6G/C could degrade most of the tested protein substrates (except for IgG) at pH 5.5 (Figures 10A,B), while pepTrCB1.6wt completely lacked such activity (not shown). Significant autodegradation of TrCB1.6G/C was noticed under the conditions of the experiment, which could to a certain degree distort the quantitative interpretation of the results.

### Identification of Hemoglobin and MBP Fragments by Mass Spectrometry

**Figure 11:**
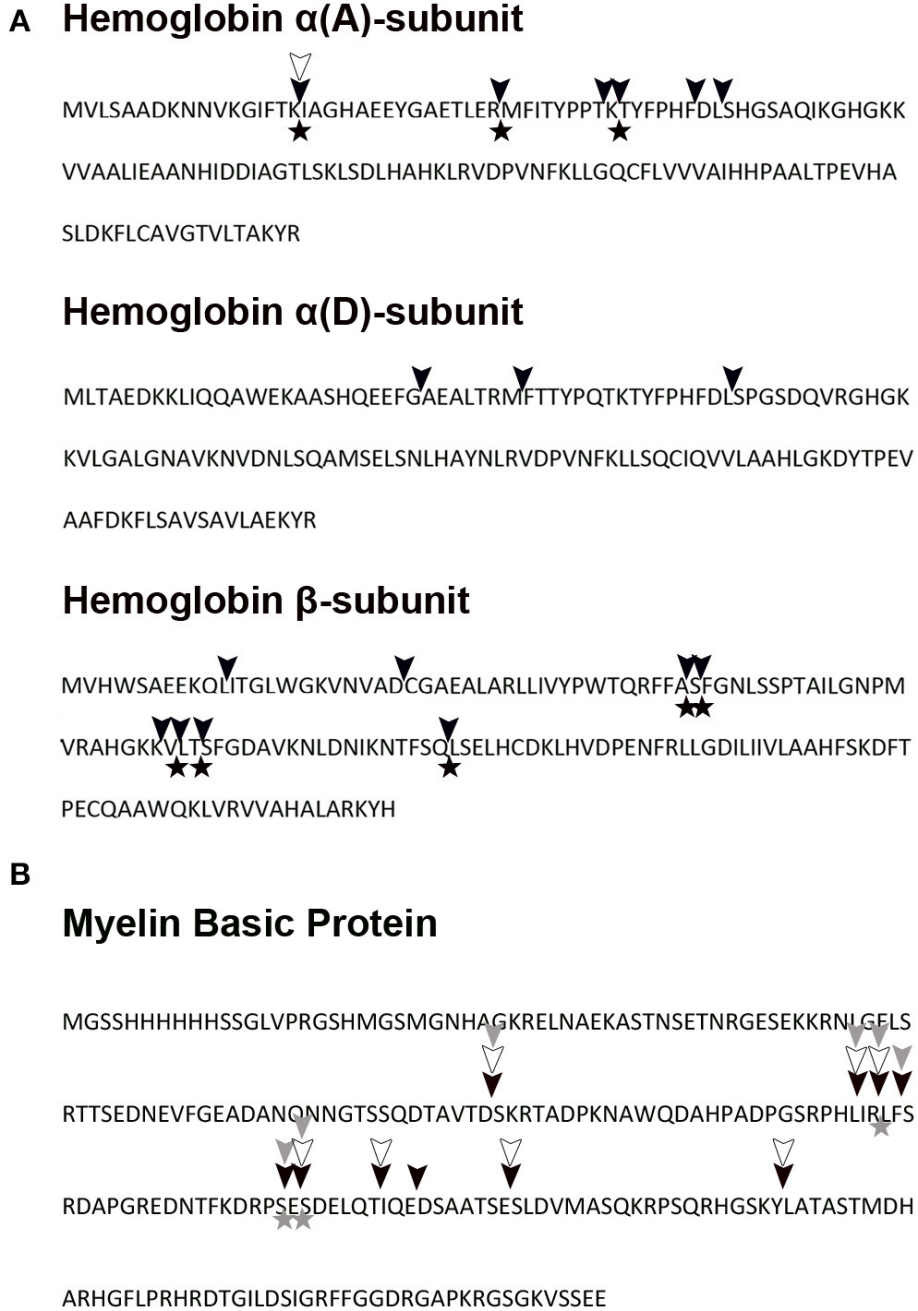
Hemoglobin and myelin basic protein pepTrCB1 cleavage map. **(A)** Turkey hemoglobin and **(B)** recombinant human myelin basic protein were digested *in vitro* with pepTrCB forms at pH 5.5. The fragments were identified by mass spectrometry, and corresponding cleavage sites are indicated in hemoglobin and myelin basic protein sequences: cleavage by pepTrCB1.1 (black triangles), cleavage by pepTrCB1.4 (empty triangles), cleavage by pepTrCB1.6G/C (gray triangles). The cleavage map combines data from digests at three time points. The initial cleavage sites (after 5 min of reaction) are marked with asterisks. Only unique fragments which were not detected at time zero are included.

The MS sequence analysis of peptides resulting from action of TrCB1 forms (pepTrCB1.1, pepTrCB1.4, and pepTrCB1.6G/C) on polypeptides of turkey Hb and human MBP was performed to determine enzymes' cleavage site specificities. Detected peptide fragments are listed in Supplementary Table 1 (only the fragments which were not detected at time zero are included). The identified cleavage sites in the sequence of α- and β-subunit of Hb and in MBP are shown in Figures 11A,B, respectively. Several fragments were obtained with pepTrCB1.1, which did not occur in controls. After 5 min of reaction, eight cleavage sites (i.e., “initial cleavage sites”) were identified. The cleavage map shows that TrCB1.1 obviously preferred Lys at P1 position in the two macromolecular substrates, followed by Arg, which corresponds with the results of PS-SCL (see above). Other amino acids could also be accommodated in this position (Leu>Thr/Glu>Phe/Ser>Gln/Ser/Gly/Met/Ala/Val/Phe). At the P2 position, the preference was not that distinctive: pepTrCB1.1 slightly preferred polar uncharged amino acids (Ser/Thr/Glu) and the positively charged Lys, closely followed by Phe/Ala/Leu/Pro/His/Asp, and less Arg/Thr/Ile/Glu. Although PS-SCL results have shown that Pro is unfavorable at P2, two of the fragments generated by pepTrCB1.1 contained Pro at this position. Only one fragment [amino acids 1 – 16 from Hbα (A) subunit] could be reliably detected after hydrolysis of Hb by pepTrCB1.4. While the same fragment generated by pepTrCB1.1 appeared after 5 min of incubation, the fragment produced by pepTrCB1.4 appeared as late as in 2 h. The preference for amino acids in both P1 and P2 positions of macromolecular substrates was not clearly defined and no obvious pattern was observed. Also, no cleavage sites distinct from those produced by TrCB1.1 were detected. Finally, no fragment was detected after incubation of Hb with TrCB1.6G/C and less fragments were also identified after cleavage of MBP comparing to the action of the other enzymes. Moreover, all the cleavage sites were identical with those produced by pepTrCB1.1 (Figure 11).

### Differential Expression of TrCB1 Forms

**Figure 12:**
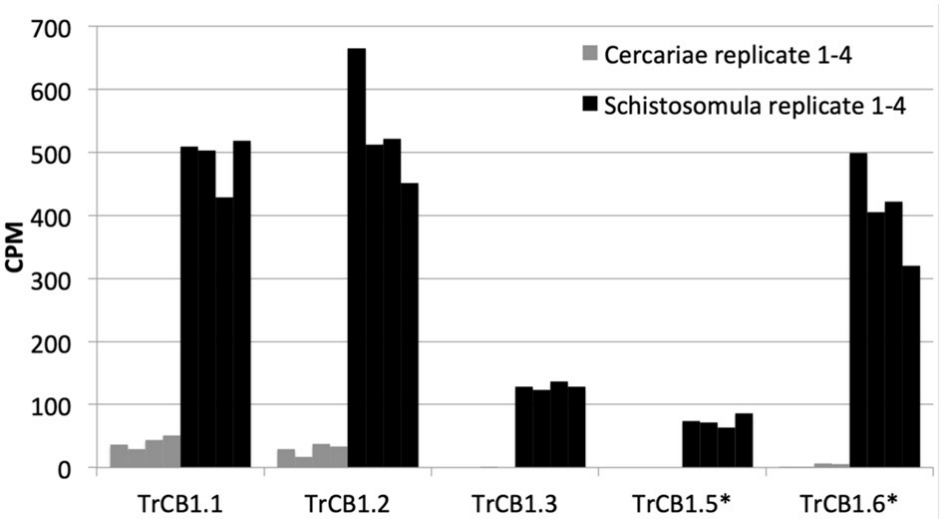
Expression profiles of TrCB1 forms. Bar charts show the level of expression of different forms of cathepsin B1 in cercariae (gray) and schistosomula (black) of *T*. *regenti* based on mapping of RNA-seq data obtained from cercariae and 7-days old schistosomula. The level of expression is expressed in counts per million (CPM) of mapped reads. Each column is a cluster of four biological replicates. Inactive paralogs are indicated by asterisks.

Based on the differential transcriptional analysis, TrCB1.1, TrCB1.2, TrCB1.3, TrCB1.5, and TrCB1.6 were stated as being significantly upregulated in schistosomula, when compared to cercariae. TrCB1.4 was not detected in *T. regenti* juvenile transcriptomes at significant levels. Transcription profile of cercariae was limited only to forms TrCB1.1, TrCB1.2, and TrCB1.6 with low counts per million (CPM) rate. In schistosomulum stage, TrCB1.1, TrCB1.2, and TrCB1.6 genes were transcribed at a similar level, followed by TrCB1.3 and TrCB1.5 (Figure 12).

### *In vitro* Exposure of Murine Astrocytes, Microglia and RAW 264.7 Macrophages to Pro-TrCB1.6wt

Production of NO by murine astrocytes, microglia and RAW 264.7 macrophages was examined after treatment with pro-TrCB1.6wt for 48 h. As revealed by Griess assay, pro-TrCB1.6wt neither triggered NO production in any of the cells nor reduced NO production in cells concurrently treated by LPS (Supplementary Figure 4A). Similarly, no effect on changes in cell viability was observed (Supplementary Figure 4B).

## Discussion

Expression of TrCB1.1 and TrCB1.4 in *Pichia* led to production of zymogens which were partly auto-processed in the yeast media, producing a small proportion of partially activated intermediates. Further processing of the recombinant (pro-) enzymes was therefore necessary. Although Dvořák et al. (2005) obtained auto-processed TrCB1.4 with an eight-fold increase in activity after overnight incubation at acidic pH, we found an insufficient increase of activity in both TrCB1.4 and TrCB1.1 using this approach. Auto-activation of recombinant cathepsin B1 from *Schistosoma mansoni* (SmCB1) is forced by negatively charged polysaccharides, such as sulfated glycosaminoglycans. Their binding to the SmCB1 pro-sequence destabilizes the pro-sequence/enzyme core junction (Jílková et al., 2014) and is mediated by a specific α-helix that contains a “heparin-binding motif”-XBBXBBX- (B is a basic amino acid and X refers to a hydropathic residue) (Horn et al., 2011; Jílková et al., 2014). A sequence alignment revealed that this motif is specific for trematode cathepsins B involved in the digestion of host proteins (Horn et al., 2011). As this motif occurs in all TrCB1 pro-sequences (Figure 2), sulfated polysaccharides facilitated auto-activation of both TrCB1.1 and TrCB1.4 (Figure 1). However, unlike in SmCB1 (Jílková et al., 2014), the products were relatively unstable at all tested pH values. Therefore, we did not use them in most subsequent experiments. However, there seems to be similarity between SmCB1 and TrCB1 in terms of glycosaminoglycan-mediated acceleration of auto-activation *in vivo*.

*In vitro* trans-processing of pro-TrCB1 by pepsin was applied according to previously published experience (Lipps et al., 1996; Law et al., 2003). N-terminal sequencing showed that the pro-sequence of TrCB1 is cleaved at carboxyterminal side of Phe and Leu residues, which is typical for pepsin (Hamuro et al., 2008). Unfortunately, none of the cleavage sites resulted in fully mature enzymes. Even prolonged incubations or higher concentrations of pepsin did not give better results. On the other hand, partial removal of the TrCB1 pro-sequences was sufficient for hydrolytic activity toward macromolecular substrates. The advantage of this method was that agarose beads-immobilized pepsin could be easily removed by centrifugation. Therefore, pepsin-processed enzymes were used in most experiments. The asparaginyl endopeptidase (AE, legumain) was confirmed as an endogenous *trans*-processing enzyme activating other digestive peptidases in human schistosomes (Sajid et al., 2003) and three transcripts coding this enzyme were also found in *T*. *regenti* transcriptome (Leontovyč et al., 2016, 2019). We were able to *trans*-process the pro-TrCB1 using tick IrAE, obtaining several intermediates during short-time incubations, but this method was not widely used in our experiments, since it provided low yields.

The substrate specificity of papain-like peptidases seems to be largely determined by the composition of the S2 pocket (Choe et al., 2006), in which the amino acid residues interact with an amino acid residue at P2 position of the peptide substrate (Schechter and Berger, 1967). From this point of view, it was interesting that amino acid preferences of TrCB1.1 and TrCB1.4 differed at the P2 position, while the preference for other positions was quite similar. Using PS-SCL, TrCB1.1 showed broader preferences at P2, having a relative preference for aliphatic amino acids (excepting Gly) over aromatic residues (Phe, Trp, and Tyr). Interestingly, TrCB1.1 had a P2 preference more similar to *S. mansoni* cathepsin B2 than to SmCB1 (Choe et al., 2006), although it has higher overall sequence identity and function similar with SmCB1. By contrast, TrCB1.4 displayed relatively narrow P2 specificity with an overriding preference for aliphatic Ala. In agreement with a previous study (Dvořák et al., 2005), TrCB1.4 did not show any noticeable activity toward substrates with Arg at P2. Surprisingly, the library assay also indicated that even Met is not a favored amino acid for TrCB1.4 at P2, which is not typical of cathepsins B (Choe et al., 2006). On the contrary, Met is the most favored amino acid accepted by TrCB1.1 at P2. Also, Z-Phe-Arg-AMC was a poor substrate for TrCB1.4, compared to TrCB1.1, wich corresponds with the results of PS-SCL.

Both TrCB1.1 and TrCB1.4 degraded a range of macromolecular substrates, such as albumin, IgG, fibrinogen, collagen type I, collagen type IV, myosin, myelin basic protein (MBP), and hemoglobin (Hb). Divergent specificities for the selected protein substrates were not observed between the two enzymes, except for MBP, which was obviously more efficiently hydrolysed by TrCB1.1. In line with previous work (Dvořák et al., 2005), both enzymes poorly degraded Hb. The authors assumed that this may indicate an adaptation to the unique migratory route of *T. regenti* through the CNS. On the other hand, Hb becomes an important source of nutrition for adult worms located in the nasal mucosa of ducks (Horák et al., 1999; Blažová and Horák, 2005; Chanová and Horák, 2007) and the level of TrCB1.1 gene transcription was comparable in *T*. *regenti* schistosomula and adults (Dolečková et al., 2010).

It is likely that active forms of TrCB1 can work in a cooperative network with other peptidases (e.g., cathepsins L and cathepsins D) in the parasite's gut, which could preferentially initiate hydrolysis of ingested host proteins (including Hb). Such a network was characterized in *S. mansoni*, where the cysteine peptidases (cathepsins L and B) initiate digestion of albumin, whereas cathepsin D is responsible for the primary cleavage of Hb, although cysteine peptidases may aid in this. Cathepsins B also act as potent (carboxy-) exopeptidases (Illy et al., 1997). Several transcripts of cathepsin L and D genes were found also in the transcriptome of *T. regenti* schistosomula (Leontovyč et al., 2016).

MS/MS analysis of protein substrate digests revealed that TrCB1.1 was more efficient in the cleavage of Hb than TrCB1.4 in terms of the number of produced cleavage sites. Nevertheless, intensive signals of undigested Hb were observed even after 2 h of incubation with the enzymes under given conditions (substrate:enzyme ratios). The cleavage sites produced by TrCB1.1 exhibited faintly defined specificity. Thus, it seems that TrCB1 enzymes may contribute to the degradation of Hb, likely after initial cleavage by other endopeptidase(s). Surprisingly, in spite of the differences in amino acid preferences at P2 of the substrates seen in PS-SCL, there was a 100% overlap in cleavage sites produced by TrCB1.4 in the molecule of MBP, with those made by TrCB1.1, which only produced a few more cuts. Unexpectedly, we were not able to detect more fragments of Hb produced by TrCB1.4, probably due to the low activity of the enzyme toward this substrate.

Although recombinant TrCB1.4 was previously obtained by employing mRNA from 5 to 6 days old schistosomula (Dvořák et al., 2005), our differential expression analysis revealed that transcripts of TrCB1.4 were not present at detectable levels in the transcriptome of cercariae or 7 days old schistosomula. This suggests that TrCB1.4 is not stably expressed in schistosomula and it is likely not essential for migrating worms in the definitive host. However, this, together with a distinct specificity toward amino acid residues of protein substrates, points to “a gene in waiting hypothesis.” The redundancy of enzyme isoforms arisen by duplication and subsequent divergence of coding genes “provides a potential mechanism to alter the substrate specificity of the protease. Such a reservoir of genes would provide protection for the parasite against host adaptation or enhancement and expansion of host range” (Salter et al., 2002). Unfortunately, data on the level of TrCB1.4 expression in adult *T*. *regenti* is not available, thus currently it can be neither excluded nor confirmed if TrCB1.4 is employed by the mature worms in order to increase the total amount or broaden the specificity of peptidases produced in the gut.

Surprising and unexpected was the relatively high expression of TrCB1 inactive paralogs, especially TrCB1.6, which was comparable to that of the active enzymes such as TrCB1.1 in the stage of schistosomulum. This raises a serious question about the importance of the catalytically inactive forms for the biology of the parasite. As expected, recombinant pro-TrCB1.6wt was incapable of auto-processing. However, the presence of an Asp residue in the pro-sequence five amino acids upstream the beginning of the mature domain indicated a potential for processing by an asparaginyl endopeptidase (AE), similarly to SmCB1 (Sajid et al., 2003) and also TrCB1.1 (Dvořák et al., 2005). This was confirmed by incubation with recombinant IrAE (Sojka et al., 2007), which resulted in a single band of ≈30 kDa formed through several intermediates arisen by specific cleavage at sites with Asn and Asp at P1 position. This suggests that the transcripts coding for AE found in the transcriptome of *T*. *regenti* (Leontovyč et al., 2016, 2019) may be involved in the removal of the pro-sequence from pro-TrCB1.6 *in vivo*. Also, trans-processing of pro-TrCB1.6wt with pepsin-processed active forms pepTrCB1.1 and pepTrCB1.4 resulted in the formation of a major (≈32 kDa) and a minor (≈30 kDa) product in both cases. The 32-kDa product was generated by cleavage between Leu^64^ and Gly^65^(pro-TrCB1.6 numbering). Unfortunately, the N-terminal sequence of the 30 kDa product could not be determined, but the size almost corresponded to that of the product obtained by IrAE processing. This data indicated that *in vivo* trans-processing of inactive forms of TrCB1 by the active ones is also possible.

For a more detailed view on the inactive TrCB1.6, a site-directed mutant TrCB1.6G/C (reverse G^29^C mutation) was expressed in *P. pastoris* system and partially characterized in comparison to the recombinant TrCB1.1 and TrCB1.4. The reverse mutation (Gly^29^ > Cys^29^) of TrCB1.6wt resulted in the restoration of hydrolytic activity toward Z-Phe-Arg-AMC, but the mutant enzyme TrCB1.6G/C was not able to cleave cathepsin B-specific substrate Z-Arg-Arg-AMC, much like TrCB1.4. This feature is caused by uncharged Ala^245^ (TrCB1.4) or Leu^245^ (TrCB1.6) at the position, where negatively charged Glu^245^ typically occurs in the S2 pocket of cathepsins B (Dvořák et al., 2005).

Moreover, TrCB16G/C activity was not inhibited by CA-074 inhibitor (mostly specific for cathepsins B). Because the occluding loop of cathepsins B is important for the interaction with CA-074 (Yamamoto et al., 1997), in which the prolyl component of CA-074 forms hydrogen bonds with the conserved His^110^ and His^111^ of the loop (bovine cathepsin numbering), the explanation may be that the latter histidine is substituted by an Asn residue in TrCB1.6, which causes the lack of susceptibility to CA-074, and also the virtual lack of exopeptidase activity. In human cathepsin B, two salt-bridge interactions between the occluding loop and the main body of the mature enzyme (Asp^22^-His^110^ and Arg^116^-Asp^224^) anchor the loop down in the active site, and unpaired His^111^ forms a salt bridge with the C-terminus of an exo-substrate (Musil et al., 1991; Illy et al., 1997). According to another study (Krupa et al., 2002), His^111^ is not critical for exopeptidase activity, although the level of this activity might be somewhat impaired by the absence of this residue. This means that also other substitutions in the sequence of TrCB1.6 are probably responsible for the disruption of exopeptidase activity in the reverse Gly^29^ > Cys^29^ mutant. The substitution Asp^224^ > Gly^224^ in the sequence of TrCB1.6, when compared to the active TrCB1 forms or other cathepsins B, seems a likely candidate, since the removal of the salt bridges between the loop and the enzyme core effectively eliminates exopeptidase activity (Illy et al., 1997).

The presence of the occluding loop was proposed to be responsible for the generally lower affinities of cathepsins B to cystatins, compared to other endopeptidases of papain-like family with open active sites (Pavlova et al., 2000). Reduced electrostatic interaction between the loop and the body of the mature TrCB1.6 might cause greater affinity to cystatin. Based on this, we hypothesized that the inactive TrCB1.6 might sequester cystatins and thus protect active TrCB1 forms or other cysteine endopeptidases present in the vicinity of this paralog from inactivation by host cystatin(s). However, repeated attempts to inhibit the peptidolytic activity of TrCB1.1 and 1.4 toward Z-Phe-Arg-AMC with chicken and human cystatins failed. Nevertheless, SDS-stable enzyme/inhibitor complexes were formed after overnight incubation of all processed TrCB1 forms with chicken cystatin, including the catalytically inactive pepTrCB1.6wt. No complex was formed with the non-processed pro-TrCB1.6wt, which suggests that the pro-sequence blocked cystatin binding to the “active site” of the paralog. Binding of chicken cystatin to TrCB1.6G/C resulted even in larger proportion of the complex and prevented the enzyme from autodegradation. Surprisingly, human cystatins B and C did not form a complex with any of the TrCB1 forms after 2 h incubation.

The TrCB1.6G/C mutant degraded most of the protein substrates similarly with TrCB1.4. It seems that the binding pockets (except for the substitution in the active site) of TrCB1.6 are intact; thereby TrCB1.6 might still preserve substrate-binding properties. Thus, the possibility that the TrCB1.6 paralog competes with the active homologs for substrates and participates in this way in post-translational regulation of the activity of these enzymes cannot be omitted.

In a previous study, active TrCB1.1 and TrCB2 were shown to trigger nitric oxide production in murine astrocyte cultures, which could be harmful to both the parasite and the host (Macháček et al., 2016). Therefore, we wanted to test whether also the TrCB1.6 paralog has any effect on the immune response of the host. We exposed immune cells naturally occurring around *T. regenti* schistosomula migrating in murine spinal cord (astrocytes, microglia, and macrophages) (Lichtenbergová et al., 2011) to recombinant TrCB1.6wt *in vitro*. Surprisingly, it neither stimulated nor inhibited nitric oxide production in the naïve or LPS-treated cells, respectively. TrCB1.6wt also did not alter the viability of these cells. Perhaps, the hydrolytic activity of TrCB1.1 and TrCB2 was somehow responsible for the above stated effect.

In conclusion, the characterization of the two active forms TrCB1.1 and TrCB1.4 showed that the enzymes differ from each other in various properties. The levels of relative expression of TrCB1 paralogs in cercariae and schistosomula indicate that both the active forms and the inactive mutants are important for schistosomula migrating and developing in the definitive host, except for TrCB1.4. This enzyme form may be expressed even later in adult worms and this supposition needs to be further verified. The ability of the TrCB1.6 paralog with non-functional, Cys-to-Gly mutated active site to bind cystatin supports the hypothesis of inhibitor sequestration, although the efficacy appeared to be low in our experimental setup. Restoration of the active site of TrCB1.6 resulted in endopeptidase but not exopeptidase activity of the paralog. This elucidated important structural features in TrCB1.6 caused by other mutations in the occluding loop and the main body of the mature enzyme, which either regulate the exopeptidase activity of cathepsin B or may also adjust the affinity of cathepsin B to cystatins. Resolving of 3-D stucture of inactive TrCB1.6 + cystatin complex could help to elucidate the nature of the binding interactions, which is also the case of TrCB1.6G/C with restored active site. Finally, the inability of TrCB1.6 paralog to affect viability and production of nitric oxide by selected immune cells does not exclude other possible interactions with host physiology/immunity, which are a subject to further investigation in this unique and fascinating neurotropic schistosome.

## Materials and Methods

### Parasite Material

*Trichobilharzia regenti* is routinely maintained in the Department of Parasitology, Charles University through domestic ducks (*Anas platyrhynchos* f. *dom*.) and lymnaeid snails (*Radix lagotis*) as definitive and intermediate hosts, respectively. Schistosomula were obtained from the spinal cord of infected ducks 9 days post-infection, washed in sterile phosphate-buffered saline (PBS) and used for RNA isolation and cDNA preparation as described previously (Dvořák et al., 2005).

Ethics statement: All procedures including animals were performed in concordance with the legislation of the Czech Republic (246/1992 and 359/2012) and the European Directive (2010/63/EU). All experiments were approved with the legal consent of the Professional Ethics Committee of the Faculty of Science, Charles University, i.e., the relevant institutional ethics committee for animal research, and of the Research and Development Section of the Ministry of Education, Youth, and Sports of the Czech Republic (approval no. MSMT-31114/ 2013-9). The animal facility, its equipment, animal welfare, and accompanying services, including the maintenance of experimental animals, have been approved by the Section of Animal Commodities of the Ministry of Agriculture of the Czech Republic (approval no. 13060/2014-MZE-17214).

### Expression and Purification of Recombinant TrCB1 Forms

Recombinant TrCB1.1 and TrCB1.4 were acquired from frozen glycerol stocks of *P. pastoris* transformants and expressed according to a previously published protocol (Dvořák et al., 2005). A column packed with Macro-Prep High S Support medium (Bio-Rad) was used for pre-purification from yeast media by cation exchange chromatography. TrCB1-containing fractions were treated with Endoglycosidase F1 (Calbiochem) following manufacturer's instructions and the pro-enzymes were further purified to homogeneity on a Mono S 5/50 GL column pre-equilibrated with 20 mM sodium acetate buffer pH 5.5 and employing 0–1 M linear NaCl gradient.

For the expression of recombinant wild-type TrCB1.6 (TrCB1.6wt), purified first-strand cDNA obtained from *T. regenti* schistosomula was used as a template for PCR with gene specific primers (Supplementary Table 2). PCR products were inserted into the expression vector pPICZα B (Thermo Fisher Scientific) and constructs were sequenced for verification (DNA Sequencing Laboratory, Faculty of Science, Charles University, Prague). To generate the Gly^29^-to-Cys^29^ mutation (TrCB1.6G/C), the pPICZα B vector containing the sequence of TrCB1.6wt was mutagenized according to the instruction in QuikChange II XL Site-Directed Mutagenesis Kit (Stratagene). The mutated vector was generated by PCR with oligonucleotide primers (each complementary to the opposite strand of the vector) containing the desired mutation (Supplementary Table 3). Following PCR, the product was treated with Dpn I endonuclease to digest methylated parental DNA template. Constructs were sequenced to verify the mutations.

The vectors containing sequences of TrCB1.6wt or TrCB1.6G/C were then electroporated into *P. pastoris* X-33 cells and recombinant proteins were produced as described above. Both TrCB1.6wt and TrCB1.6G/C were expressed with a polyhistidine affinity tag (6x His-tag) situated on the C-terminus. The proteins were purified by Ni^2+^ chelating chromatography (HisTrap^TM^FF crude, GE Healthcare Life Science) equilibrated with 100 mM phosphate buffer pH 7 with 300 mM NaCl and 40 mM imidazole. A linear gradient of 100–500 mM imidazole in the same buffer was used for elution. The eluted proteins were deglycosylated (see above) and then purified to homogeneity by cation exchange FPLC (BioLogic, Bio-Rad) on a Mono S 5/50 GL column (GE Healthcare Life Science) equilibrated with 20 mM phosphate buffer pH 7.2 and using a linear gradient of 0–1 M NaCl.

Protein concentrations were measured by Quant-iT Protein Assay Kit (ThermoFisher Scientific). Molecular size of purified enzymes was estimated by SDS-PAGE and peptidolytic activity was measured with the peptidyl substrate Z-Phe-Arg-AMC (see section Peptidase Activity). Fractions after chromatography were stored at −80°C or lyophilized.

Theoretical MW/pI values and potential N-glycosylation sites were determined by the Compute Mw software and NetNGlyc 1.0 server available at the ExPASy web portal (https://www.expasy.org/tools) (Artimo et al., 2012).

### Activation/Processing of Recombinant Enzymes

The possibilities of auto- and heterologous processing of pro-TrCB1 zymogens were investigated in order to produce mature (pro-sequence-free) fully active enzymes for subsequent analyses.

**(A)** The pro-TrCB1.1 and 1.4 zymogens (0.2 – 0.3 μg/μl) were incubated separately at 37°C for 5, 30, 60, 120, and 240 min in 50 mM sodium acetate buffer (pH 4, 4.5, 5, and 5.5) containing 2 mM DTT (Sigma Aldrich) either in the presence or absence of dextran sulfate (DS, MW 6.5–10 kDa; Sigma Aldrich) or heparin sodium salt (HSS, MW 6–30 kDa; Sigma Aldrich) at final concentrations of 10 or 20 μg/ml.

**(B)** The *trans*-processing of all recombinant pro-TrCB1 forms (0.2 – 0.3μg/μl) was catalyzed by pepsin immobilized on agarose beads (1U pepsin per 3.3 μg of recombinant pro-TrCB1; P0609 Sigma Aldrich) incubated at 37°C (pro-TrCB1.1, pro-TrCB1.4, and pro-TrCB1.6wt) or at RT (pro-TrCB1.6G/C) for 5, 30, 30, and 60 min in 50 mM sodium acetate buffer pH 4. The reaction was stopped by addition of 10 μM pepstatin A and the samples were centrifuged (1,000 × g, 5 min) to remove agarose-bound pepsin.

**(C)** The inactive pro-TrCB1.6wt was processed by recombinant asparaginyl endopeptidase of the hard tick *Ixodes ricinus*—IrAE (Sojka et al., 2007; kindly provided by Dr. Daniel Sojka, Institute of Parasitology, ASCR, Ceské Budějovice, Czech Rep.). One gram of the lyophilized yeast medium containing IrAE was dissolved in 7 ml of distilled water, concentrated and transferred to 500 μl of 50 mM sodium acetate buffer pH 4.5 containing 2 mM DTT using Amicon® Ultra 4 mL filters (MWCO 30 kDa; Millipore). IrAE was then incubated for 30 h at 37°C to allow self-processing, which was monitored fluorometrically with Z-Ala-Ala-Asn-AMC substrate (25 μM; Bachem). Subsequently, the pro-TrCB1.6wt (16.5 μg in 55 μl) was incubated with activated IrAE (5 μl, unknown concentration—see above) at 37°C in 50 mM sodium acetate buffer (pH4, 4.5, or 5) containing 2 mM DTT for 30,60, 120, and 240 min or 16 and 36 h.

**(D)** Alternatively, pro-TrCB1.6wt (0.2–0.3 μg/μl) was dissolved in 50 mM sodium acetate buffer pH 4.5 containing 2 mM DTT, 10 μM pepstatin A, and 10 μg/ml DS. Then the pepsin-activated pepTrCB1.1 or pepTrCB1.4 (2 μg/ml) was added overnight (up to 16 h) at 37°C. Controls contained 10 μM E-64.

Aliquots were taken from reaction mixtures at specified time intervals during incubations and catalytic activity of TrCB1 forms was measured with Z-Phe-Arg-AMC substrate (see section Peptidase Activity). Additionally, a shift in molecular weight was monitored by SDS-PAGE. N-terminal amino acid sequences of the proteins electroblotted onto PVDF membranes were identified by Edman degradation (Dr. Zdeněk Voburka, Laboratory of Medicinal Chemistry, IOCB, ASCR, Prague, Czech Rep.).

Active-site labeling of processed TrCB1 forms with a fluorescent probe BODIPY green-DCG-04 (Greenbaum et al., 2002; obtained from Dr. Doron C. Greenbaum, Tropical Disease Research Unit, Sandler Center for Basic Research in Parasitic Diseases, UCSF, USA—former affiliation) was carried out as described previously (Dvořák et al., 2005). The specificity of probe binding was verified by pre-incubation of controls with 10 μM irreversible cysteine peptidase inhibitor E-64 for 5 min.

### Peptidase Activity

The peptidase activity of TrCB1 forms processed as described above (0.45 μg TrCB1 per well, i.e., 70 nM) was assayed with two synthetic fluorogenic substrates (25 μM, Bachem): Z-Phe-Arg-AMC, (cathepsin L/B substrate) and Z-Arg-Arg-AMC (cathepsin B-selective substrate) in 50/100 mM citrate/phosphate buffer (CPB), 2 mM DTT, pH 3–8 (final volume 200 μl). The reactions were performed in 96-well black flat bottom plates (Nunc) using Infinite M200 fluorometer (TECAN) with excitation and emission wavelengths set to 360 and 465 nm, respectively. The release of AMC was measured at RT in a 30 min kinetic cycle at 2 min intervals.

An exopeptidase (peptidyl dipeptidase) activity assay using Bz-Gly-His-Leu as a substrate was performed with pepTrCB1 forms employing a modified protocol (Sajid et al., 2003). The pepTrCB1 forms (1 μg, 160 nM) were incubated for 15 min with the substrate in 50/100 mM CPB pH 4–5.5 containing 2 mM DTT (final volume 100 μl). Spontaneous reaction of the emerging free amino groups of His-Leu with fluorescamine (0.05 mg/ml) was monitored in a fluorometer set to 390/475 nm excitation/emission wavelengths during a 30 min kinetic cycle at 2 min intervals.

### Inhibition Assays

The effect of two inhibitors, E-64 and CA-074 (Sigma Aldrich) on endopeptidase activity of the pepTrCB1 forms (0.2 μg, 30 nM) was tested with Z-Phe-Arg-AMC. The concentrations of inhibitors ranged from 0.01 to 10 μM in the case of pepTrCB1.1 and pepTrCB1.4, and from 0.01 to 100 μM with pepTrCB1.6G/C. The samples were pre-incubated with inhibitors for 15 min prior to the measurement. Inhibition assays were carried out at the pH optima of the particular enzymes (pH 5.5 for TrCB1.1, pH 5 for TrCB1.4, and pH 6.5 for pepTrCB1.6G/C). The remaining relative enzyme activity was expressed in relation to control reactions performed in the absence of the inhibitors.

The inhibition assays of exopeptidase activity of the enzymes were performed with E-64 and CA-074 (10 μM) added prior to the substrate.

The effect of chicken egg white cystatin, human cystatin B, and human cystatin C (C8917, C4249, and H5041, Sigma Aldrich) on TrCB1 forms by was tested using dextran sulfate-activated and pepsin-activated enzymes ds/pepTrCB1.1, ds/pepTrCB1.4, ds/pepTrCB1.6G/C. Processed TrCB1 forms (0.2 μg, 30 nM) were pre-incubated with various concentrations of cystatins (0–1 μM) for 15 min prior to the measurement with 25 μM Z-Phe-Arg-AMC in the presence or absence of 2 mM DTT. All samples contained 0.01% non-ionic detergent SP BRIJ L23 (Croda).

### Formation of Peptidase-Cystatin Complex

The dsTrCB1.1, dsTrCB1.4, dsTrCB1.6G/C, and pepTrCB1.6wt (3 μg, 3.75 μM) were incubated with chicken egg white cystatin (1 μg, 3 μM) in 50/100 mM CPB pH 5.5 containing 0.01 % BRIJ L23 for 15 min at RT or overnight at 4°C. The pepTrCB1.1, pepTrCB1.4, and pepTrCB1.6 were incubated with human cystatin B and C for 2 h at RT (concentrations as above). Controls did not contain cystatins. Then the samples were mixed with non-reducing sample buffer and allowed to stand for 15 min at RT. The samples were separated by SDS-PAGE under mild conditions (80 V).

### Subsite Specificity Exploration of TrCB1.1 and TrCB1.4 by a Positional Scanning Synthetic Combinatorial Library (PS-SCL)

Purified non-processed pro-TrCB1.1 and pro-TrCB1.4 were used for the analysis of S1 – S4 subsite preferences toward P1–P4 residues of substrates. Since both pro-enzymes were able to cleave small peptide substrates, we had chosen this possibility to eliminate a potential risk of residual pepsin activity in pepsin-processed TrCB1 samples, which could bias the results. PS-SCL was applied as described previously (Choe et al., 2006). The peptidolytic activity of purified pro-enzymes (0.5 μg, 70 nM) was measured at RT in 50/100 mM CPB pH 5 containing 100 mM NaCl and 2 mM DTT.

### Hydrolysis of Protein Substrates by TrCB1 Forms

The substrates (all Sigma-Aldrich) included turkey hemoglobin (Hb, H0142), bovine myelin basic protein (MBP, M1891), myosin (M7266), human albumin (A9511), collagen I (C7774), collagen IV (C8374), IgG (I4506), and fibrinogen (F3879) at final concentrations of 0.5 mg/ml, which were incubated for 6 h with pepTrCB1 forms (1 μg) in 50 μl of 50/100mM CPB pH 5.5 containing 2 mM DTT and 10 μM pepstatin A. Control reactions did not contain the enzymes. Aliquots of 20 μl of the reaction mixtures were separated by SDS-PAGE. Optimal pH for hydrolysis was ascertained using Hb and MBP as substrates in pH range 4.5–6.5.

### Identification of Hb and MBP Fragments by Mass Spectrometry

For MALDI MS/MS analysis, turkey Hb (0.5 mg/ml) or recombinant human MBP (0.2 mg/ml, ab171675, Abcam) were incubated with 1 μg of pepTrCB1.1, pepTrCB1.4, and pepTrCB1.6G/C at 37°C in 25/50 mM CPB pH 5.5 containing 2 mM DTT and 10 μM pepstatin A, total reaction volume was 60 μl. Aliquots of 10 μl were taken after 5, 30, and 120 min and immediately treated with 0.5% trifluoroacetic acid. Control reactions did not contain the enzymes. Buffer employed for TrCB1 processing reactions that was incubated with pepsin-agarose beads was used as another control to assess possible residual activity of pepsin in the pepTrCB1-substrate samples.

MALDI-MS and MALDI-MS/MS were performed using Ultraflextreme instrument (Bruker Daltonics, Germany) operated in the linear or reflectron mode with detection of positive ions. Ferulic acid (12.5 mg/ml in water: acetonitrile: formic acid, 50:33:17 v/v mixture) or alpha-cyano-4-hydroxycinnamic acid (saturated solution in water: acetonitrile: trifluoroacetic acid, 47.5:50.0:2.5 v/v mixture), respectively, were used as the MALDI matrices. Detected peptide ions were subjected to MS/MS analysis (in a LIFT arrangement) and the resulting fragments were then searched against the primary amino acid sequences of TrCB1 forms [GenBank: AY648119 – 24], porcine pepsinogen [AAA31095.1], turkey α-A-globin [AAB35442.1], α-D-globin [AAB35443.1], β-globin [P02113.1], and human MBP (sequence available on http://www.abcam.com/recombinant-human-myelin-basic-protein-ab171675.html) using MASCOT 2.4 search engine (MatrixScience). No enzyme specificity and oxidation (M) and deamidation (N, Q) as variable modifications were set for all searches.

### Differential Expression of TrCB1 Forms

Full-length sequences of TrCB1 genes [GenBank: AY648119 – AY648124] (Dvořák et al., 2005) combined with *T. regenti* juvenile transcriptomes (cercariae and 7 days old schistosomula) (Leontovyč et al., 2016) have been used as the reference for the differential expression analysis (the data can be accessed via the NCBI website https://www.ncbi.nlm.nih.gov/ under BioProject database ID: PRJNA292737). All transcripts homologous to cathepsin B (*n* = 14) (BLASTp; E-value cut off: < 10^−5^) have been removed from *T. regenti* transcriptome and replaced by all six TrCB1 sequences from NCBI database. Trimmed and corrected Illumina reads (HiSeq 2500 platform) from *T. regenti* cercariae and schistosomula were mapped to the reference using RSEM (Li and Dewey, 2011), with zero mismatch rate (i.e., only 100% identical reads were mapped to the reference). Predicted expected counts were rounded to the highest whole number and used for differential gene transcription analysis in edgeR v.3.6.7 (Robinson et al., 2010) and R v.3.2.3 (R Development Core Team, 2015) software packages. Cathepsin B sequences with more than log_2_ fold change and false discovery rate of ≤0.01 were stated as differentially expressed between cercariae and schistosomula.

### *In vitro* Effect of Pro-TrCB1.6wt on Murine Astrocytes, Microglia and RAW 264.7 Macrophages

Primary murine astrocyte and microglia cultures were obtained from mixed glial cultures as described elsewhere (Macháček et al., 2016). These cells were used because they naturally occur and are activated in the vicinity of *T. regenti* schistosomula migrating in murine spinal cord (Lichtenbergová et al., 2011). Murine RAW 264.7 macrophages were received from Dr. Tereza Leštinová (Charles University, Prague). The cells were seeded in 96-well plates (Nunclon Delta Surface, Thermo Fisher Scientific) and exposed to 1 μg/ml of purified and deglycosylated pro-TrCB1.6wt with or without 0.5 μg/ml of lipopolysaccharide from *E. coli* 0127:B8 (LPS, Sigma Aldrich) for 48 h at 37°C. The medium itself or medium supplemented with 0.5 μg/ml of LPS was used as negative or positive control, respectively. Griess assay was used to analyze NO production by stimulated cells (Macháček et al., 2016). Cell viability after stimulation was assessed by fluorescein diacetate (Sigma Aldrich) according to manufacturer's instructions. The results were evaluated by ordinary one-way analysis of variance (ANOVA) followed by Tukey's multiple comparisons test, performed in GraphPad Prism, version 6.

## Data Availability Statement

The datasets generated for this study can be found in the NCBI BioProject ID: PRJNA292737.

## Ethics Statement

The animal study was reviewed and approved by Professional Ethics Committee of the Faculty of Science, Charles University.

## Author Contributions

HD performed expression of the enzymes, their processing, activity and inhibition assays, participated in protein purification, prepared samples for mass spectrometry, participated in interpretation of experimental data and preparation of figures, and wrote a larger part of the manuscript. RL obtained transcriptomic data, processed and interpreted the data on differential enzyme expression. TM performed immunological experiments and interpreted the data. AO'D and HD obtained and interpreted PS-SCL data. OŠ and ZZ obtained and interpreted mass spectrometry data. CSC provided know-how, facility and material for PS-SCL experiments, and performed critical reading of the whole manuscript. CRC and HD performed site-directed mutagenesis in TrCB1.6. AO'D and CRC provided critical comments on technical performance and interpretation of the results. PH revised the manuscript critically for important intellectual content. LM prepared conception, coordinated experiments, participated in protein purification, and cystatin-binding assays, contributed to interpretation of experimental data and finalized the manuscript. All authors contributed to writing or finalization of the manuscript.

### Conflict of Interest

The authors declare that the research was conducted in the absence of any commercial or financial relationships that could be construed as a potential conflict of interest.
