# Supplementary material for: Isoforms of Cathepsin B1 in Neurotropic Schistosomula of Trichobilharzia regenti Differ in Substrate Preferences and a Highly Expressed Catalytically Inactive Paralog Binds Cystatin
Source: Front Cell Infect Microbiol. 2020 Feb 26;10:66. doi: 10.3389/fcimb.2020.00066 (PMC7054455; doi:10.3389/fcimb.2020.00066)
Supplement: Supplementary file 1 [file Data_Sheet_1.PDF]

Supplementary Figure 1. SDS-PAGE of purified deglycosylated pro-TrCB1 forms

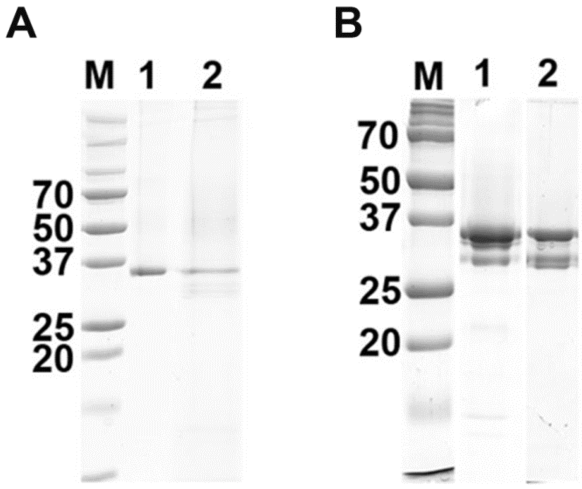

(A) Lane 1, recombinant pro-TrCB1.6wt (2 µg). Lane 2, pro-TrCB1.6G/C (2 µg). **(B)** Lane 1, pro-TrCB1.1 (5 µg). Lane 2, pro-TrCB1.4 (5 µg). Pro-enzymes were treated by endoglycosidase F1. M, markers of molecular size.
