# Supplementary material for: Isoforms of Cathepsin B1 in Neurotropic Schistosomula of Trichobilharzia regenti Differ in Substrate Preferences and a Highly Expressed Catalytically Inactive Paralog Binds Cystatin
Source: Front Cell Infect Microbiol. 2020 Feb 26;10:66. doi: 10.3389/fcimb.2020.00066 (PMC7054455; doi:10.3389/fcimb.2020.00066)
Supplement: Supplementary file 2 [file Data_Sheet_2.PDF]

Supplementary Figure 2. Self-processing of TrCB1.1 and TrCB1.4 under acidic conditions

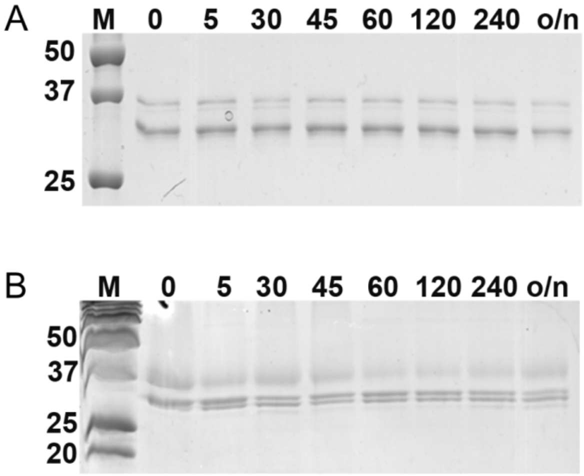

The attempts to fully auto-activate the pro-enzymes at low pH were largely unsuccessful. The gels show samples of **(A)** (pro-)TrCB1.1 and **(B)** (pro-)TrCB1.4 in pH 4 taken at time intervals indicated above the gel (in min). A proportion of the enzymes was processed during preceding steps of purification (lower bands). o/n, overnight incubation.
