# Supplementary material for: Isoforms of Cathepsin B1 in Neurotropic Schistosomula of Trichobilharzia regenti Differ in Substrate Preferences and a Highly Expressed Catalytically Inactive Paralog Binds Cystatin
Source: Front Cell Infect Microbiol. 2020 Feb 26;10:66. doi: 10.3389/fcimb.2020.00066 (PMC7054455; doi:10.3389/fcimb.2020.00066)
Supplement: Supplementary file 3 [file Data_Sheet_3.PDF]

Supplementary Figure 3. Trans-processing of pro-TrCB1.6wt by *Ixodes ricinus* asparaginyl endopeptidase at various pH values.

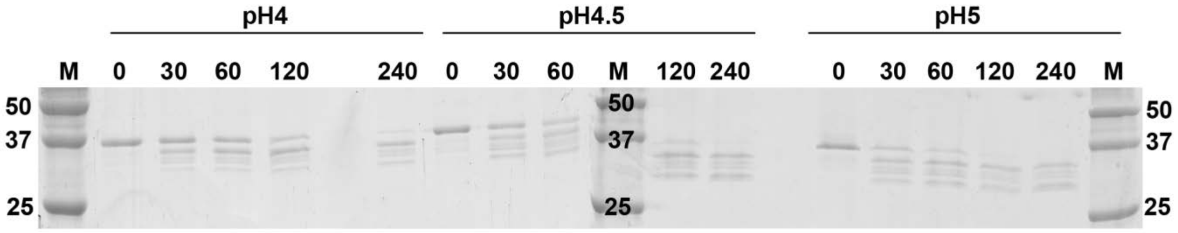

The pro-TrCB1.6wt was incubated at three different pH values with purified yeast medium containing activated IrAE for various time periods at 37 °C. The TrCB1.6wt processing products were resolved by SDS-PAGE. M, markers of molecular size (kDa).
