# Supplementary material for: Isoforms of Cathepsin B1 in Neurotropic Schistosomula of Trichobilharzia regenti Differ in Substrate Preferences and a Highly Expressed Catalytically Inactive Paralog Binds Cystatin
Source: Front Cell Infect Microbiol. 2020 Feb 26;10:66. doi: 10.3389/fcimb.2020.00066 (PMC7054455; doi:10.3389/fcimb.2020.00066)
Supplement: Supplementary file 4 [file Data_Sheet_4.PDF]

Supplementary Figure 4. Pro-TrCB1.6wt did not affect production of nitric oxide (NO) or viability of astrocytes, microglia or RAW 264.7 macrophages.

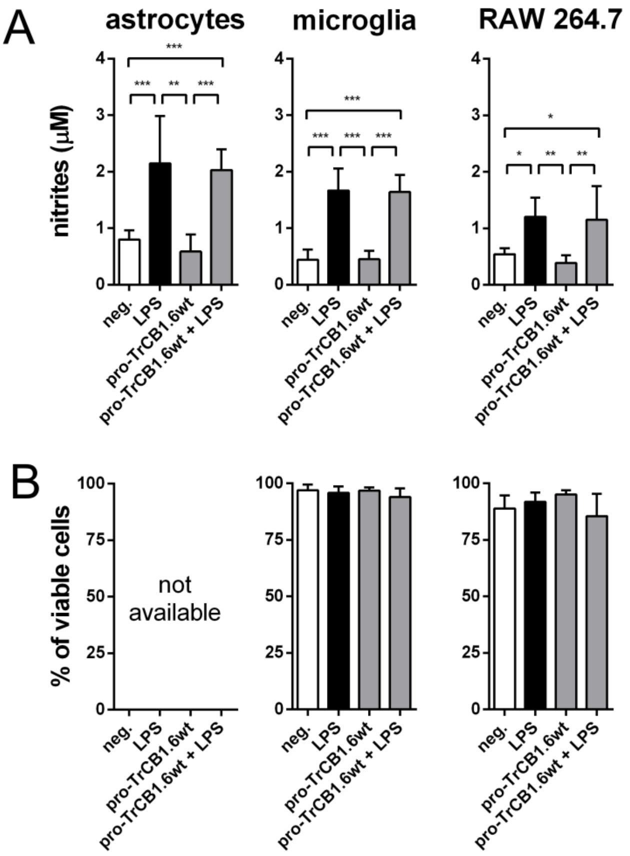

(A) Primary astrocytes, primary microglia and RAW 264.7 macrophages were treated with LPS (0.5 μg/ml) and/or pro-TrCB1.6wt (1 μg/ml). After 48 hours, production of nitric oxide and cell viability were examined by Griess assay or fluorescein diacetate staining, respectively. pro-TrCB1.6wt neither increased NO production nor reduced it in cells co-treated with LPS. **(B)** No detrimental effects on cell viability were noticed. As for astrocytes, the data on viability are not available since the cells in all examined groups had always detached before the analysis was finished. However, no morphological changes suggesting altered physiological status were noticed in any of the treated groups. Data (n = 6) were evaluated by one-way ANOVA followed by Tukey's test (* $p<0.05$, **$p<0.01$, *** $p<0.001$).
