# Supplementary material for: Isoforms of Cathepsin B1 in Neurotropic Schistosomula of Trichobilharzia regenti Differ in Substrate Preferences and a Highly Expressed Catalytically Inactive Paralog Binds Cystatin
Source: Front Cell Infect Microbiol. 2020 Feb 26;10:66. doi: 10.3389/fcimb.2020.00066 (PMC7054455; doi:10.3389/fcimb.2020.00066)
Supplement: Supplementary file 5 [file Data_Sheet_5.PDF]

Supplementary Table 1. Identified peptides resulting from the action of TrCB1 forms on hemoglobin and myelin basic protein

Peptidase	Section	Identified peptide	m/z	Substrate (subunit)
TrCB1.1	1 - 16	VLSAADKNNVKGIFTK-I*	1704.9	Hb α (A)
	17 - 31	K-IAGHAEEYGAETLER-M*	1645.8	Hb α (A)
	17 - 39	K-IAGHAEEYGAETLERMFITYPPT-K**	2596.2	Hb α (A)
	17 - 40	K-IAGHAEEYGAETLERMFITYPPTK-T*	2724.3	Hb α (A)
	17 - 46	K-IAGHAEEYGAETLERMFITYPPTKTYFPHF-D***	3516.8	Hb α (A)
	17 - 48	K-IAGHAEEYGAETLERMFITYPPTKTYFPHFDL-S**	3744.9	Hb α (A)
	1 - 25	MLTAEDKKLIQQAWEKAASHQEEFG-A, oxidized**	2904.5	Hb α (D)
	33 - 48	M-FTTYPQTKTYFPHFDL-S**	2006.0	Hb α (D)
	1 - 10	VHWSAEEKQL-I**	1226.6	Hb β
	44 - 66	A-SFGNLSSPTAILGNPMVRAHGKK-V**	2382.3	Hb β
	44 - 67	A-SFGNLSSPTAILGNPMVRAHGKKV-L*	2481.3	Hb β
	45 - 66	S-FGNLSSPTAILGNPMVRAHGKK-V*	2295.2	Hb β
	45 - 67	S-FGNLSSPTAILGNPMVRAHGKKV-L*	2394.3	Hb β
	70 - 87	T-SFGDAVKNLDNIKNTFSQ-L*	1998.0	Hb β
	90 - 114	D-SKRTADPKNAWQDAHPADPGSRPHL-I**	2752.7	MBP
	117 - 135	R-LFSRDAPGREDNTFKDRPS-E***	2208.2	MBP
	117 - 136	R-LFSRDAPGREDNTFKDRPSE-S**	2337.3	MBP
	117 - 142	R-LFSRDAPGREDNTFKDRPSESDELQT-I**	3010.7	MBP
	119 - 145	F-SRDAPGREDNTFKDRPSESDELQTIQE-D**	3120.7	MBP
	153 -171	E-SLDVMASQKRPSQRHGSKY-L**	2175.2	MBP
TrCB1.4	1 - 16	VLSAADKNNVKGIFTK-I***	1704.9	Hb α (A)
	90 - 114	D-SKRTADPKNAWQDAHPADPGSRPHL-I***	2752.7	MBP
	117 - 136	R-LFSRDAPGREDNTFKDRPSE-S**	2337.3	MBP
	117 - 142	R-LFSRDAPGREDNTFKDRPSESDELQT-I**	3010.7	MBP
	153 -171	E-SLDVMASQKRPSQRHGSKY-L***	2175.2	MBP
TrCB1.6G/C	90 - 118	D-SKRTADPKNAWQDAHPADPGSRPHLIRLF-S**	3281.9	MBP
	117 - 135	R-LFSRDAPGREDNTFKDRPS-E*	2208.2	MBP
	117 - 136	R-LFSRDAPGREDNTFKDRPSE-S*	2337.3	MBP

* Fragments obtained after 5 min of incubation. ** Fragments obtained after 30 min of incubation. *** Fragments obtained after 2 h of incubation. Cleavage sites in peptide sequences are indicated by hyphens (-). Hb, hemoglobin; MBP, myelin basic protein.
