# Supplementary material for: Isoforms of Cathepsin B1 in Neurotropic Schistosomula of Trichobilharzia regenti Differ in Substrate Preferences and a Highly Expressed Catalytically Inactive Paralog Binds Cystatin
Source: Front Cell Infect Microbiol. 2020 Feb 26;10:66. doi: 10.3389/fcimb.2020.00066 (PMC7054455; doi:10.3389/fcimb.2020.00066)
Supplement: Supplementary file 6 [file Data_Sheet_6.PDF]

Supplementary Table 2. Specific primers used for amplification of TrCB1.6wt gene.

Primer name	Primer sequence
TrCB1.6F	5´TCTC/TCGAG*AAAAGA*AATGAGATGCAATTCGAACCC 3´
TrCB1.6R	5´ATGC/GGCCGCTCA**ATGATGATGATGATGATG**ATTACGAGGTAATCCACCTAT 3´

XHOI, NOTI restriction sites are underlined, Kex 2 peptidase cleavage site *initalic*, termination codon shaded in grey, 6His-tag **in bold.**
