# Supplementary material for: Isoforms of Cathepsin B1 in Neurotropic Schistosomula of Trichobilharzia regenti Differ in Substrate Preferences and a Highly Expressed Catalytically Inactive Paralog Binds Cystatin
Source: Front Cell Infect Microbiol. 2020 Feb 26;10:66. doi: 10.3389/fcimb.2020.00066 (PMC7054455; doi:10.3389/fcimb.2020.00066)
Supplement: Supplementary file 7 [file Data_Sheet_7.PDF]

Supplementary Table 3. Primers for site-directed mutagenesis of TrCB1.6.

Primer name	Primer sequence
TrCB1.6G/CF	5´ CAATCTCGATGTGGTTCATGCTGGGCATTCGCTGCAGTTG 3´
TrCB1.6G/CR	5´ CAACTGCAGCGAATGCCCAGCATGAACCACATCGAGATTG 3´

Substitution of nucleotides G/C for A/T shaded in grey.
